# The Uridylyl Transferase TUT7‐Mediated Accumulation of Exosomal miR‐1246 Reprograms TAMs to Support CRC Progression

**DOI:** 10.1002/advs.202304222

**Published:** 2024-02-11

**Authors:** Yifei Feng, Chi Jin, Tuo Wang, Zhihao Chen, Dongjian Ji, Yue Zhang, Chuan Zhang, Dongsheng Zhang, Wen Peng, Yueming Sun

**Affiliations:** ^1^ Department of General Surgery The First Affiliated Hospital with Nanjing Medical University Nanjing Jiangsu 210029 P. R. China; ^2^ The First School of Clinical Medicine Nanjing Medical University Nanjing 210029 China

**Keywords:** colorectal cancer, exosomes, NLRP3, tumor‐associated macrophages, TUT7

## Abstract

Tumor‐associated macrophages (TAMs) play a crucial role in promoting tumor growth and dissemination, motivating a search for key targets to interfere with the activation of TAMs or reprogram TAMs into the tumor‐suppressive type. To gain insight into the mechanisms of macrophage polarization, a designed co‐culture system is established, allowing for the education of macrophages in a manner that closely mimics the intricacies of TAMs in the tumor immune microenvironment (TIME). Through database mining, exosomal miR‐1246 is identified and is then validated. Exosomal miR‐1246‐driven polarization of TAMs disrupts the infiltration and function of CD8^+^ T cells. Mechanically, the amassment of exosomal miR‐1246 stems from TUT7‐mediated degradation of small noncoding RNA, a process stabilized by SNRPB, but not the precursor of miR‐1246. Moreover, an Exo‐motif is present in the exosomal miR‐1246 sequence, enabling it to bind with the exosomal sorting protein hnRNPA2B1. RNA‐seq analysis reveals that exogenous miR‐1246 modulates the polarization of TAMs at a post‐transcriptional level, emphasizing the pivotal role of the NLRP3 in macrophage polarization. In conclusion, the findings underscore the importance of exosomal miR‐1246 as a trigger of macrophage reprogramming and uncover a novel mechanism for its enhanced presence in the TIME.

## Graphical abstract

Tumor cells release exosomal miR‐1246, which reprograms macrophages toward a tumor‐supportive type. This alteration modulates specific signaling within the macrophages, promoting tumor growth and hindering CD8^+^ T cell infiltration.

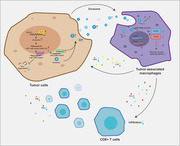

## 1 Introduction

Colorectal cancer (CRC) remains one of the most common malignant tumors in China, with increased incidence in recent years.^[^ ^1^ ^]^ Surgical intervention is currently the main therapeutic approach for CRC, yet for patients with advanced or metastatic cases, it often fails to yield satisfactory outcomes,^[^ ^2^, ^3^ ^]^ since as many as 30% of patients experience a recurrence or metastasis in the long term after surgical treatment.^[^ ^4^ ^]^

Recently, immunotherapy has emerged as a promising alternative treatment, offering hope for patients with little surgical benefit.^[^ ^5^, ^6^, ^7^ ^]^ However, the lack of effective therapy targets has thus far hindered its widespread adoption. At the core of immunotherapy lies the concept of the tumor immune microenvironment (TIME), which provides a “fertile soil” for cancer progression. Immune cells, particularly tumor‐associated macrophages (TAMs), or T cells, constitute a critical component of TIME, through the exchange and transmission of substances and information with adjacent cancer cells.^[^ ^8^, ^9^ ^]^

Studies have shown that TAMs regulate tumor cell proliferation and metastasis.^[^ ^10^ ^]^ Conventionally, macrophages are divided into two phenotypes: the classically activated M1 type and the selectively activated M2 type.^[^ ^11^ ^]^ In the TIME, most macrophages exhibit a tumor‐supporting type, resembling the M2 type. While there are similarities between M2 macrophages and TAMs, evidence suggests that they are not identical at the genetic or phenotypic level.^[^ ^12^ ^]^ TAMs are known to secrete various growth factors that promote cancer development, such as the CHI3L1 protein, which drives metastasis in breast cancer and regulates tumor cell metabolism through PGK1 in glioma.^[^ ^13^, ^14^ ^]^

Additionally, TAMs are involved in modulating the infiltration and function of CD8+ T cells.^[^ ^15^ ^]^ Thus, identifying targets to alter the activation or reprogramming of TAMs into the M1 phenotype holds great potential for enhancing tumor immunotherapy. The polarization of macrophages is shaped by an intricate interplay between cancer cells and macrophages. In lung cancer, tumor cells modulate macrophage polarization by secreting FGF2 and M‐CSF.^[^ ^16^, ^17^ ^]^ Similarly, in CRC, PKN2 in tumor cells has been shown to activate the Erk1/2 pathway, triggering macrophage polarization.^[^ ^18^ ^]^

Moreover, small extracellular vesicles, such as exosomes, are also critical mediators of cell‐to‐cell communication, predominantly between tumor cells and macrophages.^[^ ^19^, ^20^ ^]^ Several studies have demonstrated that microRNA‐enriched exosomes secreted by tumor cells regulate macrophage polarization in cancers.^[^ ^21^, ^22^ ^]^ miR‐1246 has been found to be upregulated in serum samples and exosomes from CRC patients.^[^ ^23^, ^24^, ^25^ ^]^ Cooks et al. have suggested that mutant p53 may enhance tumor‐supporting macrophages by increasing the levels of exosomal miR‐1246;^[^ ^26^ ^]^ however, the source of exosomal miR‐1246 in CRC cells and its mechanism in macrophages remain unclear. Our previous research showed that forced METTL3 expression increases miR‐1246 levels in cancer cells,^[^ ^27^ ^]^ but uncertainty still exists on whether METTL3 contributes to exosomal miR‐1246 in CRC.

In the present study, we conducted a comprehensive investigation of the role of exosomal miR‐1246 in mediating macrophage polarization in CRC. Our findings reveal that exosomal miR‐1246 skews macrophages toward a tumor‐supporting type. Moreover, we found that TUT7 and hnRNPA2B1 synergistically upregulated the expression of exosomal miR‐1246 in a manner dependent on RNU2‐1 degradation. Furthermore, the upregulation of exogenous miR‐1246 in macrophages regulated NLRP3 expression at the post‐transcriptional level. These findings allow us to gain deeper insight into the mechanisms of macrophage polarization and present a novel approach for therapeutic applications in CRC.

## 2 Results

### 2.1 Cancer Cells Reprogram Macrophages to a Tumor‐Supportive Type

**Figure 1:**
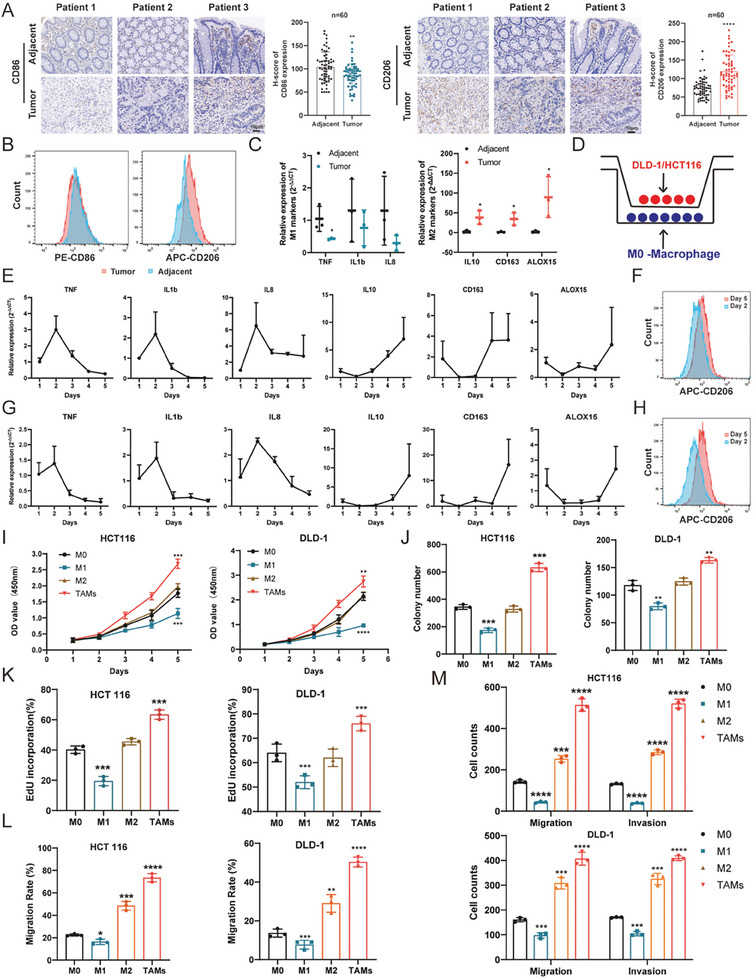
Cancer cells reprogram macrophages to a tumor‐supporting type. A) Immunochemistry analysis of macrophage markers (CD86 for M1 macrophages and CD206 for M2 macrophages) in CRC patients (*n* = 60). Representative images and corresponding statistical results (H‐score) are shown. Scale bars = 10 µm. B) Flow cytometry of CD206 in macrophages isolated from tumor and adjacent normal tissues. Three pairs of samples were analyzed. C) qPCR analysis of macrophages isolated from the tumor and adjacent normal tissues (M1 macrophage markers: TNF, IL1b, and IL8; M2 macrophage markers: IL10, CD163, and ALOX15). Three pairs of samples were analyzed. D) Schematic representation of the transwell system used for induction of TAMs in vitro. M0 macrophages were seeded in the lower chamber while CRC cells resided in the upper chamber. E–G) Relative mRNA expression of macrophage markers (TNF, IL1b, and IL8 for M1 macrophages; IL10, CD163, and ALOX15 for M2 macrophages) in the coculture system with THP1‐derived macrophages and DLD‐1 cells (upper panel) /HCT116 cells (lower panel) at each time point, *n* = 3. F–H) Flow cytometry of CD206 in macrophages induced by cancer cells, assessed at specific time points as indicated (day 2 vs day 5). I–K) Proliferation analysis of DLD‐1 and HCT116 cells, cultured with CM from M0, M1, M2, and TAMs (induced macrophages at day 5), as assessed by CCK‐8, colony formation, and Edu assays. Statistical results were shown. Three biological replicates were analyzed. L,M) Metastatic analysis of cocultured tumor cells, determined by the transwell and wound healing assays. Statistical results are displayed. Three biological replicates were analyzed. Bar graphs show the mean ± SD. */**/***/****p < 0.05/0.01/0.001/0.0001.

To confirm the subtype distribution of macrophages in CRC, immunohistochemical analysis of macrophage markers indicated that M2 macrophages (CD206 positive) were highly present in tumor tissues, whereas M1 macrophages (CD86 positive) were scarce (**Figure** **1**A). Subsequently, TAMs were isolated from both adjacent normal and tumor tissues and analyzed by flow cytometry, which revealed a marked difference in subtype distribution, with elevated levels of CD206 and decreased CD86 in tumors (Figure 1B). This was further validated by qPCR, which demonstrated the upregulation of M2 markers in the tumor tissues (Figure 1C).

Previous studies have shown that cancer cells can induce the phenotypic transition of macrophages to a tumor‐supportive type in lung cancer in vitro.^[^ ^28^ ^]^ To replicate this phenomenon, M0 macrophages were cocultured with the DLD‐1 and HCT116 cells for 5 days using a transwell apparatus (Figure 1D). The cancer cells were replaced daily to maintain maximal stimulation. Analysis of macrophage marker gene expression demonstrated that the majority of M1 genes (TNF, IL‐1B, and IL‐8) were upregulated in the first 2 days and then downregulated, whereas M2 genes (IL‐10, CD163, and ALOX15) were downregulated in the first 2 days and then upregulated (Figure 1E and Figure S1A, Supporting Information). Flow cytometry confirmed that relative to day 2 coculture, macrophages on day 5 showed higher levels of CD206 (Figure 1F and Figure S1B, Supporting Information).

To validate these findings, human monocytes isolated from peripheral blood mononuclear cells (PBMCs) were used. These PBMC‐derived macrophages were cocultured with the conditioned medium (CM) extracted from DLD‐1 and HCT116 cells, following the aforementioned protocol. qPCR and flow cytometry analyses confirmed similar results (Figure 1G,H and Figure S1C,D, Supporting Information). For this reason, macrophages induced by cancer cells on day 5 were selected for the functional assessment. The CM of macrophages on day 5 was extracted and then incubated with HCT116 and DLD‐1 cells for 24 h, and M0, M1 (classically activated), and M2 (alternatively activated) macrophages were used as controls. Notably, cells cocultured with the CM from macrophages on day 5 showed a significant increase in proliferation (Figure 1I–K and Figure S1E,F, Supporting Information) and migration abilities (Figure 1L,M and Figure S1G,H, Supporting Information).

Although M1 macrophages effectively suppressed the proliferation and migration of CRC cells, M2 macrophages had slight or no impact on cell functions (Figure 1I–M and Figure S1E–I, Supporting Information). Thus, we obtained macrophages induced by cancer cells on day 5 as M2‐like TAMs, which closely resemble the TAMs in TME in vivo, and were used as the TAMs throughout this study. Also, these findings may highlight two key points. First, although there are some similarities between M2‐like TAMs (TAMs) and traditional M2 macrophages, particularly in terms of macrophage marker gene expression, they are not identical, and cancer‐cell‐induced macrophages better mimic the TAMs in vitro than traditional M2 macrophages. Second, CRC cells can reprogram macrophages to a tumor‐supportive phenotype.

### 2.2 Polarization of Macrophages by Exosomal miR‐1246

**Figure 2:**
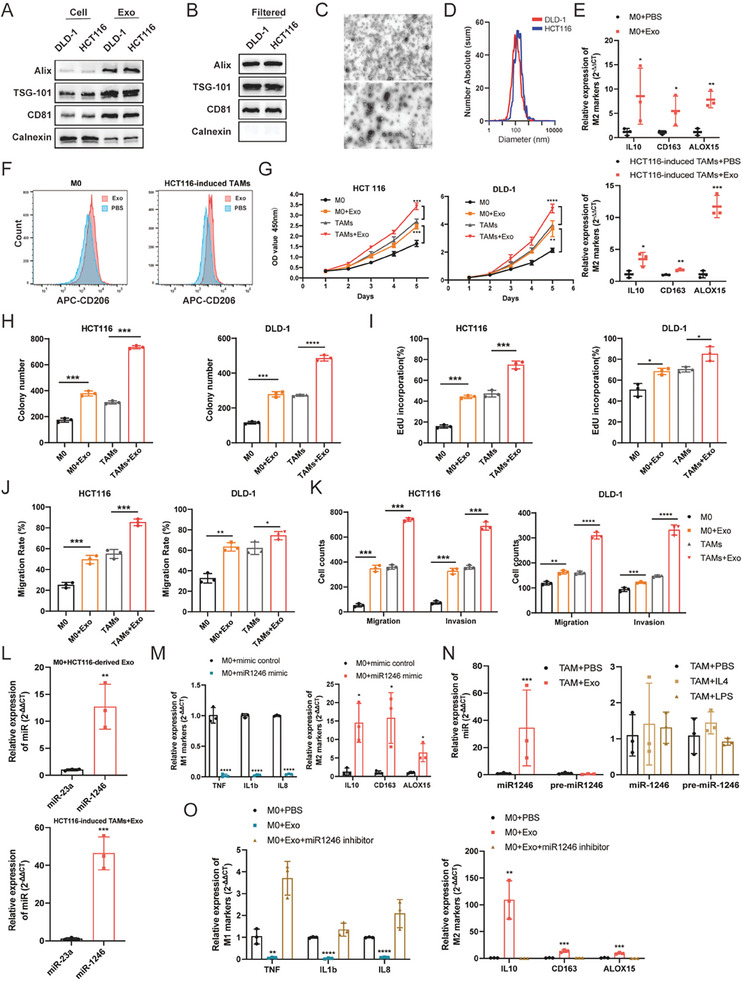
Polarization of macrophages by exosomal miR‐1246. A) Western blot analysis of exosomal markers from DLD‐1 and HCT116 cell‐derived exosomes including Alix, TSG‐101, and CD81, with Calnexin as a cellular contaminant marker. B) Exosomes filtered (0.22 µm) to exclude any cellular contamination were confirmed by western blot. C) Transmission electron microscopy (TEM) of paraformaldehyde (PFA)‐fixed exosomes isolated from DLD‐1 (upper panel) and HCT116 (lower panel) cells supernatant, with representative images shown. Scale bars = 1 µm. D) The size distribution of exosomes was determined by nanoparticle tracking analysis (NTA). E) The mRNA level of M2 marker genes in M0 macrophages (upper panel) and HCT116‐induced TAMs (lower panel), treated with cancer‐derived exosomes or control PBS. F) Flow cytometry analysis of CD206 marker in M0 macrophages and HCT116‐induced TAMs after treating with HCT116‐derived exosomes or control PBS, the fluorescence intensity was displayed. G–I) CCK‐8, colony formation, and EdU analyses of DLD‐1 and HCT116 cells cultured with CM from M0 and TAMs, both pre‐treated with or without HCT116‐derived exosomes. The statistical analysis is presented; *n* = 3 biological replicates. J,K) Migration and invasion assays of DLD‐1 and HCT116 cells cultured with CM from M0 and TAMs with or without HCT116‐derived exosomes, and the statistical analysis is presented; *n* = 3 biological replicates. L) qPCR analysis of miR‐1246 and miR‐23a levels in M0 macrophages (upper panel) and HCT116‐induced TAMs (lower panel), which were pretreated with HCT116‐derived exosomes. M) The expression of M1 and M2 marker genes in M0 macrophages transfected with miR‐1246 scramble or mimics. N) The levels of miR‐1246 and pre‐miR‐1246 in TAMs (HCT116 cell induced) cultured with exosomes, IL4, LPS, or PBS. O) M0 macrophages cultured with HCT116‐derived exosomes, followed by transfection with miR‐1246 inhibitors, and then the expression of M1 and M2 marker genes was assessed by qPCR. Bar graphs show the mean ± SD. */**/***/****p < 0.05/0.01/0.001/0.0001.

Cancer cells, without a direct connection, were capable of reprogramming macrophages in TIME. This led to the hypothesis that exosomes secreted by CRC cells participated in this process. Indeed, exosomes, particularly those from cancer cells or immune cells, are deeply involved in many intercellular interactions including regulation of the TIME and formation of pre‐metastatic niches.^[^ ^29^ ^]^ To test this, exosomes were isolated and characterized from the supernatant of HCT116 and DLD‐1 cells. Immunoblot analysis with TSG‐101, CD81, and Alix confirmed the presence of exosomal markers (**Figure** **2**A). Any cellular contamination was excluded (0.22 µm filter) before use (Figure 2B). The extracted exosomes were further verified for size distribution and morphology through transmission electron microscopy (TEM) and nanoparticle tracking analysis (NTA) (Figure 2C,D).

To assess the effect of exosomes on macrophage polarization, M0 macrophages and cell‐induced TAMs were treated with exosomes extracted from HCT116 cells, and phosphate‐buffered saline (PBS) was designated as a control. As expected, the expression of M2 markers increased significantly in exosome‐treated macrophages, compared to the control group (Figure 2E and Figure S2A, Supporting Information). These results were further supported by flow cytometry analysis, which revealed an increase in the number of positive CD206‐stained cells (Figure 2F and Figure S2B, Supporting Information). To determine the role of exosome‐exposed macrophages in CRC cell development, we evaluated the proliferation and migration of CRC cells in vitro. Results indicated that exosome‐treated macrophages enhanced proliferation (Figure 2G–I and Figure S2C,D, Supporting Information) and migration (Figure 2J,K and Figure S2E,F, Supporting Information) of CRC cells, compared to the control group. These findings confirmed that exosomes derived from CRC cells modulate macrophage polarization in vitro.

MicroRNAs (miRNAs) are among the abundant particles encapsulated in exosomes,^[^ ^30^ ^]^ and several circulating exosomal miRNAs have been shown to be upregulated in CRC samples. A previous miRNA profile of exosomes revealed that 16 miRNAs were significantly upregulated in both patient samples and cell line media, with 8 of these being downregulated after primary tumor resection (Table S1, Supporting Information).^[^ ^23^ ^]^ Another study showed that the serum levels of 18 miRNAs increased significantly with liver metastasis (Table S1, Supporting Information).^[^ ^31^ ^]^ Integrated analysis of these datasets (Figure S2G, Supporting Information) and subsequent qPCR analysis of serum samples from CRC patients and healthy donors confirmed that miR‐23a and miR‐1246 were expressed at higher levels in exosomes from cancer samples, compared to controls (Figure S2H, Supporting Information). Additionally, we detected higher levels of miR‐1246 in induced TAMs compared to miR‐23a (Figure S2I, Supporting Information), which was also observed in exosome‐treated M0 macrophages and exosome‐treated TAMs (Figure 2L and Figure S2J, Supporting Information). Therefore, we postulate that miR‐1246 may be a critical mediator in this process. Indeed, treatment of M0 macrophages with miR‐1246 mimics enhanced the expression of M2 marker genes, while simultaneously downregulating the levels of M1 marker genes (Figure 2M and Figure S2K, Supporting Information). Moreover, we noticed that the level of miR‐1246 in exosome‐treated TAMs increased significantly, without any changes in pre‐miR‐1246 expression, while IL‐4 or lipopolysaccharide (LPS) treatment had no impact on the expression of either miR‐1246 or pre‐miR‐1246 in these cells (Figure 2N and Figure S2L, Supporting Information), suggesting that the increase in miR‐1246 in TAMs is predominantly due to exogenous sources. Additionally, we extracted exosomes from HCT116 and DLD‐1 cells and used them to polarize M0 macrophages for 24 h. Then, miR‐1246 inhibitors were transfected into the induced macrophages to deplete miR‐1246 expression. The subsequent qPCR results demonstrated that exosomes from both DLD‐1 and HCT116 cells facilitated M2 polarization of M0 macrophages, and these effects were diminished by miR‐1246 inhibitors (Figure 2O and Figure S2M, Supporting Information). Therefore, the exosomal miR‐1246 originating from CRC cells mediates the polarization of macrophages in CRC.

### 2.3 Macrophages Induced by miR‐1246 Promote CRC Progression

**Figure 3:**
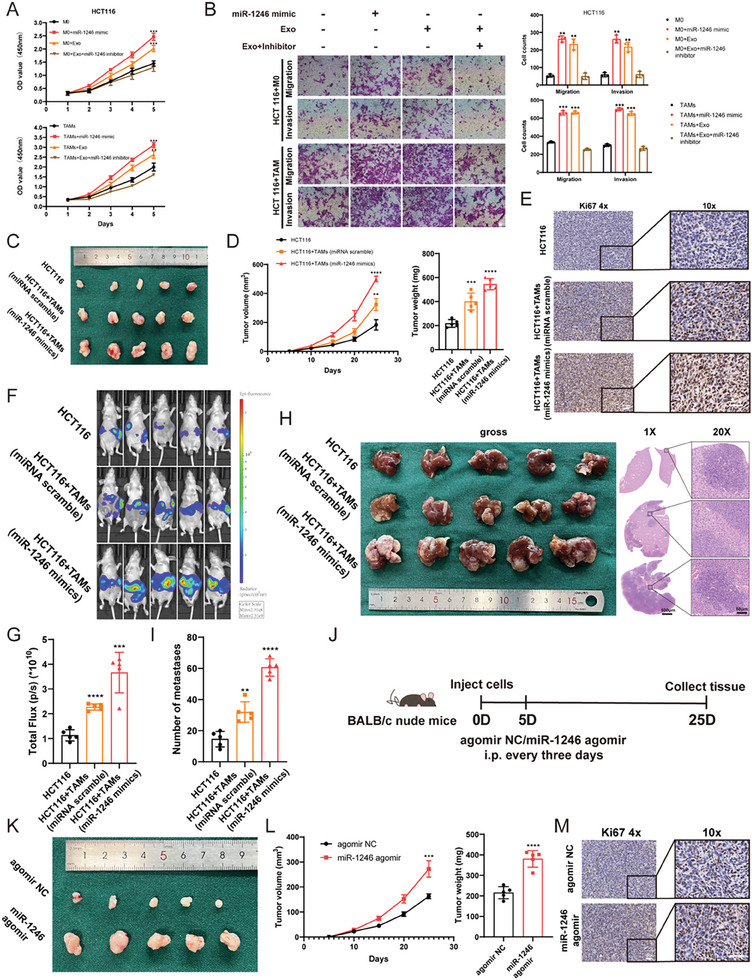
Macrophages Induced by miR‐1246 Promote CRC Progression. A,B) CCK‐8 and transwell analyses of HCT116 cells cultured with CM from M0 macrophages and TAMs transfected with exosomes, miR‐1246 mimics, and exosomes with miR‐1246 inhibitors for 24 h. C) Gross pictures of a subcutaneous tumor model of HCT116 cells cocultured with TAM transfected with miRNA mimics or scramble, *n* = 5. D) Statistical quantification of subcutaneous tumors in HCT116 cells with the indicated treatments. E) Immunostaining of Ki67 in representative tumors with the indicated treatments, *n* = 5. Scale bars = 100 µm. F) Representative images of the luminescence intensity of the tumor metastasis of HCT116 cells co‐cultured with TAMs transfected with miRNA mimics or scramble, *n* = 5. G) Relative quantification of the luminescent fluxes of tumor metastatic model. H) Gross images of metastatic tumors and the representative H&E‐stained sections in the livers. I) Statistical result of the number of metastatic nodules in the livers. J) Schematic of the procedure for the injection of miR1246 agomir and agomir NC in the subcutaneous tumor model. K) The development of subcutaneous tumors in mice with orthotopic injection of HCT116 cells and treatment of miR‐1246 agomir. L) Statistical results of tumor volume (left panel) and tumor weight (right panel) in miR‐1246 agomir loading mice. M) Ki‐67 immunostaining of the sections in miR‐1246 agomir loading mice, *n* = 5. Scale bars = 100 µm. Bar graphs show the mean ± SD. */**/***/****p < 0.05/0.01/0.001/0.0001.

Since the phenotypic transition of macrophages regulates the proliferation, migration, and invasion capabilities of cancer cells, we sought to investigate the effects of miR‐1246‐induced macrophages on the development of CRC. To do this, we utilized CM collected from both M0 macrophages and TAMs that had been polarized by exosomes with miR‐1246 mimics and exosomes with miR‐1246 inhibitors. These CM were then cocultured with HCT116 cells and the proliferation and migration abilities of HCT116 were determined using the CCK‐8 and Transwell assays, respectively. As expected, both exosomes and miR‐1246‐reprogrammed macrophages enhanced the cancer cell biological malignant behavior, while miR‐1246 inhibitors possessed the opposite effects (**Figure** **3**A,B and Figure S3A,B, Supporting Information). To further validate these findings in vivo, two different mouse models were deployed. In the first model, miR‐1246‐mimics or scramble‐treated TAMs were cocultured with HCT116 cells, followed by injection into a) the flanks of nude mice to establish the subcutaneous tumors; or b) the spleen tips of nude mice to initiate metastatic liver tumors. Untreated HCT116 cells were used as a control. The results showed that the mimics‐induced group developed significantly larger xenograft tumors compared to the control and miRNA scramble groups in vivo (Figure 3C–I). Further analysis of the isolated macrophages from tumor samples revealed that M2 marker genes were significantly upregulated in the miR‐1246 mimics group, while M1 marker genes were downregulated (Figure S3C, Supporting Information). Flow cytometry analysis also indicated an increase in the amount of M2 macrophages in the miR‐1246 mimics group (Figure S3D, Supporting Information).

In the second mouse model, a chemically modified miRNA agonist, miR‐1246 agomir, which possesses high stability and activity in vivo, was administrated. Prior to the injection, a subcutaneous tumor model was established (Figure 3J). Similar to previous findings, the miR‐1246 agomir significantly promoted primary tumor growth (Figure 3K–M). Macrophages were isolated 3 weeks later, and qPCR analysis showed an increase in M2 marker genes and a corresponding decrease in M1 markers (Figure S3E, Supporting Information). Moreover, the number of CD206‐positive macrophages increased in the miR‐1246 mimics group (Figure S3F, Supporting Information). Taken together, these results suggest that exosomal miR‐1246 facilitates the polarization of TAMs, thereby promoting the proliferation, migration, and invasion of cancer cells.

### 2.4 RNU2‐1 Contributes the Accumulation of Exosomal miR‐1246

**Figure 4:**
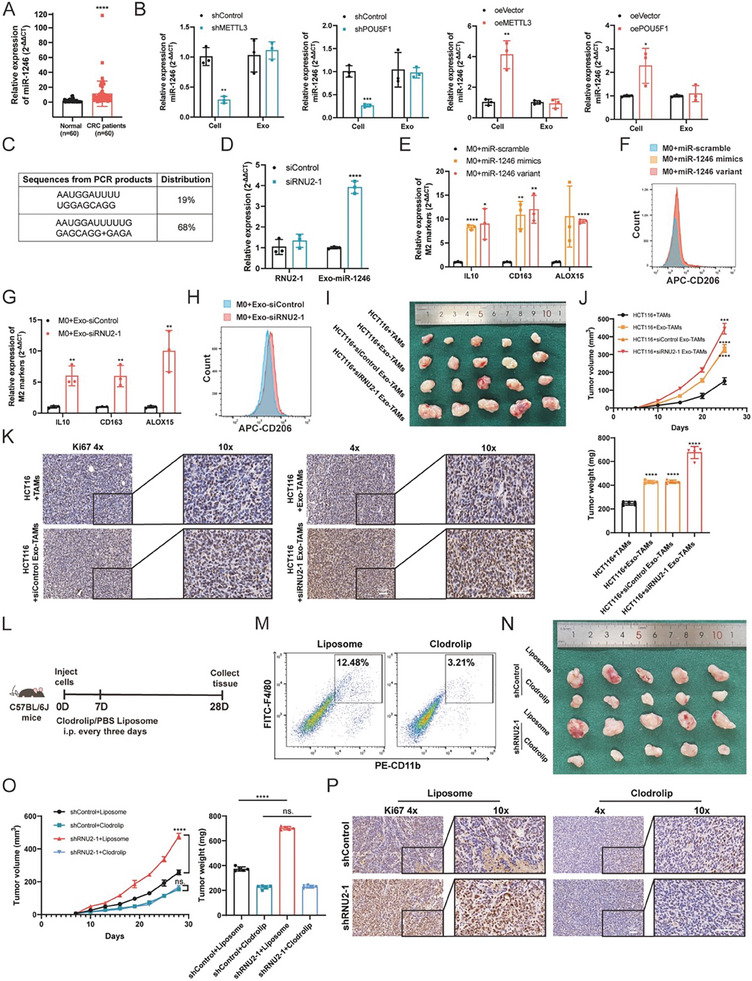
RNU2‐1‐derived exosomal miR‐1246 Contribute to Macrophage Polarization. A) qPCR analysis of miR‐1246 level in the serum of CRC patients, *n* = 60. B) Relative expression of cellular and exosomal miR‐1246 in the presence of shMETTL3, oeMETTL3, shPOU5F1, and oePOU5F1 in HCT116 cells, measured by qPCR. C) The percentage distribution of the sequencing results from PCR products in HCT116‐derived exosomes. D) Expression of RNU2‐1 and exosomal miR‐1246 in HCT116 cells with siRNU2‐1 and normal control. E) Levels of M2 marker genes in M0 macrophages transfected with RNU2‐1f (miR‐1246 variant), miR‐1246 mimics, and scramble control. F) Flow cytometry of CD206 in M0 macrophages transfected with RNU2‐1f, miR‐1246 mimics, and scramble control. G) The levels of M2 marker genes in M0 macrophages induced by exsomes from HCT116 cells harboring siRNU2‐1 or normal control. H) The count of CD206 positive cells in M0 macrophages treated with exosomes from HCT116 cells harboring siRNU2‐1 or normal control. I) Gross pictures of a subcutaneous tumor model in HCT116 cells coultured with pretreated TAMs, including PBS control, exosomes from the cancer cells, and exosomes from siRNU2‐1 transfected cancer cells, *n* = 5. J) Statistical quantification of subcutaneous tumors in HCT116 cells coultured with pretreated TAMs, including PBS control, exosomes from the cancer cells, and exosomes from the siRNU2‐1 transfected cancer cells. K) The immunostaining of Ki67 in representative tumor sections in (I), bars = 100 µm. L) Schematic of the strategy for macrophage depletion in subcutaneous mice model. M) Flow cytometry quantified the CD206 positive macrophages in HCT116 xenografts following treatment with Clodrolip or liposome control. N,O) Gross pictures and statistical results in HCT116 xenografts following treatment with Clodrolip or the liposome control. HCT116 cells were transfected with shRNU2‐1 or normal control before the inoculation, *n* = 5. P) The immunostaining of Ki67 in representative tumor sections treated with Clodrolip versus the liposome control. Bar graphs show the mean ± SD. */**/***/****p < 0.05/0.01/0.001/0.0001.

Although the upregulation of miR‐1246 in various cancers has been well documented, the relationship between its enrichment in exosomes and levels of POU5F1 (Oct4) and METTL3 has yet to be established.^[^ ^32^, ^33^ ^]^ qPCR analysis of miR‐1246 in the serum of CRC patients demonstrated an elevated level (**Figure** **4**A), and although the higher expression of POU5F and METTL3 was confirmed from The Cancer Genome Atlas (TCGA) database and CRC samples (Figure S4A,B, Supporting Information), no clear correlation was found between the cellular expression of POU5F1/METTL3 and the serum miR‐1246 level (Figure S4C, Supporting Information). Moreover, the knockdown of these genes in HCT116 cells failed to alter the level of exosomal miR‐1246, with partial inhibition of cellular miR‐1246. Similar regulatory patterns could also be observed in the overexpression models (Figure 4B and Figure S4D,E, Supporting Information). In this regard, the precursor of miR‐1246 (pre‐miR‐1246) was overexpressed in HCT116 cells, while the overexpression only altered cellular miR‐1246 levels partially, the expression of exosomal miR‐1246 was not significantly altered (Figure S4F, Supporting Information). These findings prompt us to speculate that upregulated exosomal miR‐1246 may not be derived from mature miR‐1246 or its precursor.

Studies have argued the origins of miR‐1246, with evidence supporting the theory that circulating miR‐1246 is a degradation byproduct of U2 small nuclear RNA (RNU2‐1), rather than its precursor. To determine the origin of exosomal miR‐1246 in CRC, a polyA tailing SYBR method was applied to quantify miR‐1246 level in exosomes, and the products were cloned and sequenced. The majority of amplicons contained the RNU2‐1 fragments containing additional GAGA nucleotides (69%) but sequences complementary to the precursor of miR‐1246 could not be detected (Figure 4C), consistent with previous findings.^[^ ^34^ ^]^ We introduced siRNA to knock down RNU2‐1 level in CRC cells, but due to the constitutive expression of RNU2 transcript in eukaryotic cells, it was challenging to suppress RNU2‐1 expression using siRNA. Nevertheless, the accumulation of exosomal miR‐1246 emerged (Figure 4D), further supporting the notion that the degradation of RNU2‐1 underlies the enrichment of exosomal miR‐1246 in CRC.

To probe the functional role of this miR‐1246 variant, an expression plasmid of the miR‐1246 variant was constructed and transfected into M0 macrophages. Similar to miR‐1246 mimics, the miR‐1246 variant upregulated the expression of M2 marker genes (Figure 4E,F). To further examine the transfer of the miR‐1246 variant from tumor cells to macrophages, exosomes were isolated from CM of siRNU2‐1 or control cells and incubated with M0 macrophages. The results of qPCR and flow cytometry analysis indicated that exosomes from HCT116 cells harboring siRNU2‐1 greatly increased the levels of M2 marker genes (Figure 4G,H). Furthermore, the ability of the miR‐1246 variant to polarize macrophages was tested in vivo. Exosomes were first extracted from HCT116 cells transfected with siRNU2‐1 or normal control. These polarized macrophages were then mixed with wild‐type HCT116 cells in a 1:1 ratio and subcutaneously injected into BALB/c nude mice to form a xenograft tumor model. The macrophages induced by exosomes from RNU2‐1‐knockdown cells possessed a potent impact on the proliferation of cancer cells (Figure 4I–K). Moreover, the level of M2 markers and distribution of CD206‐positive cells were significantly altered in macrophages isolated from the tumor mass (Figure S4G,H, Supporting Information).

To determine whether the effects of the miR‐1246 variant relied upon macrophages in vivo, clodronate liposome (Clodrolip) was used to eliminate macrophages in mice. First, lentivirus‐mediated RNU2‐1 shRNA (shRNU2‐1) and control vector were obtained and transfected with MC38 cells. An in vitro functional assay confirmed that shRNU2‐1 had no impact on cell proliferation (Figure S4I, Supporting Information). A subcutaneous tumor model with C57BL/6J mice was established and treated with Clodrolip intraperitoneally (IP) every 3 days to deplete macrophages after 1 week of inoculation (Figure 4L). As expected, Clodrolip decreased the infiltration of TAMs in the TIME (Figure 4M). Notably, shRNU2‐1 successfully accelerated tumor growth, but this effect was abrogated by Clodrolip treatment (Figure 4N–P). These results indicate that exosomal miR‐1246 derived from RNU2‐1 can modulate macrophage polarization both in vitro and in vivo.

### 2.5 TUT7 Facilitates the Degradation of RNU2‐1

**Figure 5:**
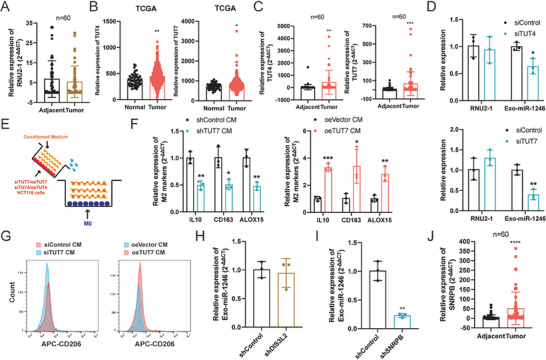
TUT7 facilitates the accumulation of exosomal miR‐1246 in CRC. A) The level of RNU2‐1 was assessed in CRC patients, *n* = 60. B) The expression patterns of TUT4 and TUT7 were analyzed in the TCGA database. C) TUT4 and TUT7 expressions were detected in CRC patients, *n* = 60. D) The levels of cellular RNU2‐1 and exosomal miR1246 in TUT4 (upper panel) or TUT7 (lower panel) knockdown cells. E) Schematic of the incubation system between CM from HCT116 cells transfected with TUT7/TUT4 knockdown or overexpression plasmids and M0 macrophages. F) The level of M2 marker genes in M0 macrophages induced by the CM from TUT7 knockdown or overexpression cancer cells. G) Flow cytometry of CD206 in M0 macrophages induced by CM from TUT7 knockdown or overexpression cells. H) Relative exosomal‐miR‐1246 level in DIS3L2‐knockdown HCT116 cells. I) Relative exosomal‐miR‐1246 level in SNRPB‐knockdown HCT116 cells. J) The expression of SNRPB was detected in CRC patients, *n* = 60. Bar graphs show the mean ± SD. */**/***/****p < 0.05/0.01/0.001/0.0001.

We noticed no discernible difference in RNU2‐1 levels between normal and cancer tissues in CRC (**Figure** **5**A). Given RNU2's critical role in eukaryotic cells, we investigated the degradation process of RNU2‐1. Current evidence supports that 5′ or 3′ end modifications regulate small nuclear RNA (snRNA) decay, with 3′ end uridylylation being the most important mediator of RNU2‐1 degradation.^[^ ^35^, ^36^ ^]^ TUT1, TUT4, and TUT7, three uridylylation enzymes that are broadly expressed in human cells, are required for snRNA decay, with TUT4 and TUT7 predominantly mediating U2 snRNAs degradation.^[^ ^37^ ^]^

To determine the presence of uridylylation in RNU2‐1 degradation, we first evaluated the expression patterns of TUT4 and TUT7 from the TCGA database and found that both enzymes were significantly upregulated (Figure 5B), which was confirmed in patient samples by qPCR analysis (Figure 5C). Upon TUT4 or TUT7 knockdown in HCT116 cells, the RNU2‐1 level remained unchanged; however, the level of exosomal miR‐1246 decreased significantly (Figure 5D). Interestingly, TUT7 knockdown resulted in greater reductions in exosomal miR‐1246 compared to TUT4 knockdown (Figure 5D), suggesting that a higher abundance of TUT7 in tumors should be the main reason for the accumulation of miR‐1246 in exosomes. To test this, we manipulated TUT7 and TUT4 expression in HCT116 cells and collected the corresponding CM (Figure 5E and Figure S5B, Supporting Information). The CM from TUT7‐overexpressing cells polarized macrophages to a tumor‐supporting type, as evident by increased levels of M2 markers (Figure 5F). Conversely, TUT7 knockdown decreased polarization (Figure 5F). Flow cytometry analysis concurred with these findings (Figure 5G). However, manipulating TUT4 expression in HCT116 cells did not mirror the results (Figure S5C,D, Supporting Information).

Previous research has demonstrated that DIS3L2, a 3′–5′ exonuclease, decays polyuridylated snRNAs in the cytoplasm,^[^ ^38^ ^]^ whereas TUT4 and TUT7 are primarily involved in monouridylation and/or oligouridylation. Indeed, the depletion of DIS3L2 in HCT116 cells did not affect the accumulation of exosomal miR‐1246, suggesting that the uridylated RNU2‐1 is not the substrate for DIS3L2 (Figure 5H and Figure S5E, Supporting Information). Given that RNU2‐1 shares a Sm site‐like sequence (AUUUUU),^[^ ^37^, ^39^ ^]^ we investigated the role of specific motif binding protein (SNRPB) in stabilizing miR‐1246 variants. SNRPB knockdown in HCT116 cells resulted in decreased exosomal miR‐1246 (Figure 5I and Figure S5F, Supporting Information), implying that SNRPB protects the variants from further degradation by exonucleases. Interestingly, higher expression of SNRPB in tumors was revealed in CRC samples and the TCGA database (Figure 5J and Figure S5G, Supporting Information). Taken together, our results show that upregulated TUT7 catalyzes RNU2‐1 uridylation, which in turn facilitates RNU2‐1 decay and increases exosomal miR‐1246 level in CRC.

### 2.6 HnRNPA2B1 Sorts the miR‐1246 Variant into Exosomes

Similar to exosomal protein loading, exosomal miRNA is selectively sorted and controlled by several RNA binding proteins that target a specific short sequence in miRNAs, known as the Exo‐motif.^[^ ^40^, ^41^ ^]^ To examine this, a biotinylated miR‐1246 variant was constructed and analyzed via the RNA‐protein pull‐down assay, the results of which were subjected to mass spectrometry. The top ten RNA‐binding proteins are listed in Table S2, Supporting Information. Among them, hnRNPA2B1 has previously been shown to possess this miRNA‐secretion ability by sorting miRNA into exosomes, and the miR‐1246 variant harbors this recognition motif.

**Figure 6:**
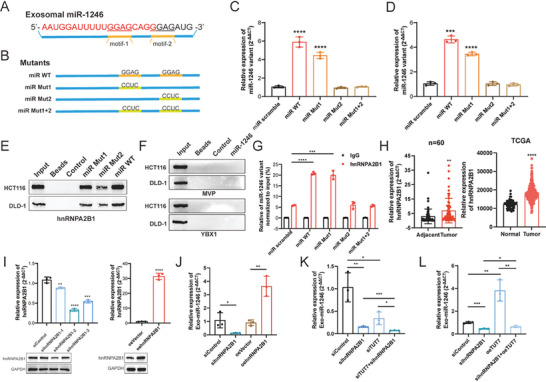
hnRNPA2B1 sorts miR‐1246 variant into exosomes in TIME. A) The exosomal miR‐1246 variant harbored two GGAG motifs at the 3′ end. B) The expression plasmids were constructed according to the mutation patterns in the sequence of the exosomal miR1246 variant. C) The level of exosomal miR‐1246 was quantified in HCT116 cells transfected with miR scramble, miR WT, miR Mut1, miR Mut2, and miR Mut1+2. D) The level of miR‐1246 in M0 macrophages induced by CM from HCT116 cells transfected with miR scramble, miR WT, miR Mut1, miR Mut2, and miR Mut1+2. E,F) Western blot analysis of the products immunoprecipitated by different miR‐1246 mutants. hnRNP2AB1, MVP, and YBX1 were tested. G) RIP assay of hnRNP2AB1 followed by qPCR of miR‐1246 in HCT116 cells transfected with different miR‐1246 mutants. H) The upregulation of hnRNPA2B1 in patient samples and TCGA cohort. I) The knockdown, and overexpression efficiency of hnRNPA2B1 in HCT116 cells was assessed by qPCR and western blot. J) The level of exosomal miR‐1246 was determined in HCT116 cells with sihnRNPA2B1 or oehnRNPA2B1. K) The level of exosomal miR‐1246 in HCT116 cells was determined in the presence of dual knockdown of TUT7 and hnRNPA2B1. L) The level of exosomal miR‐1246 in HCT116 cells transfected with oeTUT7, sihnRNPA2B1, and oeTUT7 followed by sihnRNPA2B1. Bar graphs show the mean ± SD. */**/***/****p < 0.05/0.01/0.001/0.0001.

The GGAG core motif is found to be over‐represented in many exosomal miRNAs.^[^ ^40^ ^]^ Further assessment indicated that two GGAG motifs (motif‐1 and motif‐2) exist in the miR‐1246 variant at the 3′ end (**Figure** **6**A). To identify which one or both regulate miR1246 sorting, equal amounts of scramble miRNA (miR scramble), miR‐1246 variant (miR WT), motif‐1‐mutated miR‐1246 (miR Mut1), motif‐2‐mutated miR‐1246 (miR Mut2), and the double mutants of miR‐1246 (miR Mut1+2) were expressed in HCT116 cells (Figure 6B). Subsequently, the level of exosomal miR‐1246 was quantified by qPCR. Results showed that a high amount of exosomal miR‐1246 was present in HCT116 cells treated with miR‐WT and miR‐Mut1, while miR scramble and mutants containing motif‐2 (miR Mut2, miR Mut1+2) resulted in lower levels (Figure 6C). Furthermore, when the CM from HCT116 cells transfected with these plasmids was cocultured with M0 macrophages, qPCR analysis of the miR‐1246 level in macrophages revealed that CM from the miR Mut1 and miR WT groups possessed the highest stimulatory effects (Figure 6D), suggesting that the GGAG in motif‐2 mostly accounted for the differential sorting of miR‐1246. Additionally, the RNA pull‐down performed using biotinylated miR‐1246 in HCT116 cells indicated that miR‐1246 harboring the motif‐2 immunoprecipitated hnRNPA2B1 (Figure 6E), while the involvement of MVP and YBX1 in miRNA sorting was not detected (Figure 6F). The RIP‐qPCR analysis confirmed that miR‐1246 was highly enriched in HCT116 cells transfected with miR WT and miR‐Mut1, but not other mutants (Figure 6G). Similar to TUT7, hnRNPA2B1 was overexpressed in CRC tumors (Figure 6H) and knockdown of hnRNPA2B1 in HCT116 cells significantly decreased the level of exosomal miR‐1246, whereas overexpression of hnRNPA2B1 promoted it (Figure 6I,J). In addition, dual knockdown of TUT7 and hnRNPA2B1 was significantly superior in suppressing exosomal miR1246 levels in HCT116 cells compared to individual knockdown (Figure 6K). Overexpression of TUT7 alone had little effect on exosomal miR‐1246 levels in the absence of hnRNPA2B1 (Figure 6L), indicating that the regulatory role of TUT7 depends on hnRNPA2B1. These findings suggest that the upregulation of hnRNPA2B1 plays a role in sorting miR‐1246 into exosomes in CRC.

### 2.7 Exogenous miR‐1246 Targets the NOD‐Like Receptor Pathway in TAMs

**Figure 7:**
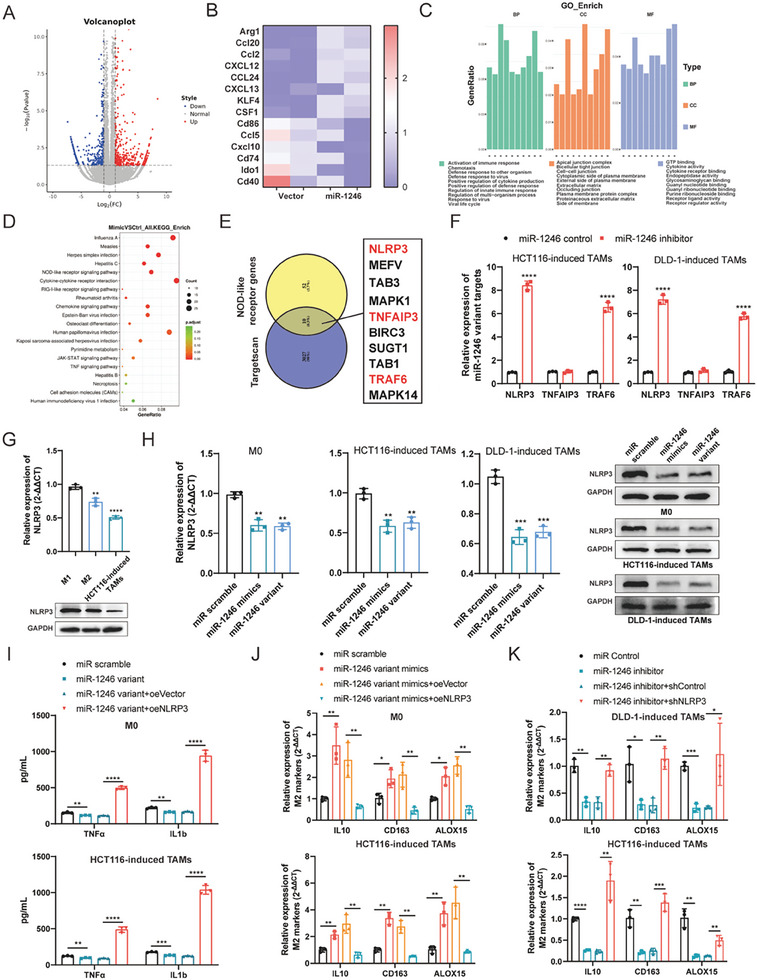
Exogenous miR‐1246 targets NOD‐like receptor pathway to regulate TAM polarization. A) Volcano plot indicating differentially expressed genes (DEGs) in TAMs transfected with miR‐1246 variant or scramble control, *n* = 2. B) The heatmap of M1 and M2 marker genes in TAMs transfected with miR‐1246 variant or scramble control. C,D) Gene ontology (GO) and Kyoto Encyclopedia of Genes and Genomes (KEGG) enrichment analysis of DEGs identified in (A). E) Venn diagram showing the overlap of target predictions of miR‐1246 and NOD‐like receptor genes. NLRP3, TNFAIP3, and TRAF6 were identified. F) Relative expression of NLRP3, TNFAIP3, and TRAF6 in TAMs transfected with miR‐1246 inhibitors. G) The level of NLRP3 in M1, M2 macrophages, and HCT116‐induced TAMs determined by qPCR and western blot. H) The level of NLRP3 in M0 macrophages and TAMs transfected with miR‐1246 mimics or miR‐1246 variant determined by qPCR and western blot. I) ELISA of TNFα and IL1β in M0 macrophages (upper panel) and HCT116‐induced TAMs (lower panel) transfected with miR‐1246 variant or miR‐1246 variant followed by oeNLRP3. J) The expression of M2 marker genes in M0 macrophages (upper panel) and HCT116‐induced TAMs (lower panel) transfected with miR‐1246 variant or miR‐1246 variant followed by oeNLRP3. K) The expression of M2 marker genes in TAMs transfected with miR‐1246 inhibitors or miR‐1246 inhibitors followed by shNLRP3. Bar graphs show the mean ± SD. */**/***/****p < 0.05/0.01/0.001/0.0001.

To elucidate mechanisms of exosomal miR‐1246 in mediating macrophage polarization, RNA‐Seq analysis was performed in TAMs transfected with miR‐1246 variant plasmid and control vector. Differently expressed genes (DEGs) consisted of 438 transcripts upregulated and 485 downregulated, as shown in **Figure** **7**A and listed in Table S3, Supporting Information. Further analysis indicated that miR‐1246 overexpression decreased the levels of inflammatory macrophage markers (Cxcl10), along with a downregulation of a M1 signature (Cd86, Ccl5, Cd74, Ido1, Cd40), while pro‐tumorigenic M2 polarization markers (Arg1, Ccl20) were strongly upregulated (Figure 7B). Nevertheless, the levels of some macrophage makers exhibited an anomalous expression pattern (Figure S6A, Supporting Information), further supporting the intricacy of TAMs in the TIME. Subsequently, the gene ontology (GO) analysis revealed enrichment for terms such as “activation of immune response,” “chemotaxis,” “cytokine activity,” and “cytokine receptor binding,” all potentiating macrophage polarization (Figure 7C). Consistently, the KEGG analysis indicated that the cytokine‐cytokine receptor interaction and NOD‐like receptor signaling pathway were enriched (Figure 7D). Moreover, JAK‐STAT and TNF signaling pathways were identified, which may be the downstream signaling pathways of NOD‐like receptors. Thus, our RNA‐seq data depicted that the miR‐1246 variant was capable of manipulating macrophage polarization via specific pathways.

To identify the downstream targets of the miR‐1246 variant, we used the Targetscan tool and identified 3037 transcripts containing at least one conserved miR‐1246 binding site in the 3′ UTR (Table S4, Supporting Information). As the NOD‐like receptor signaling pathway regulates the immune response and favors macrophage polarization into the M1 state,^[^ ^42^ ^]^ the predictions overlapped with KEGG NOD‐like receptor signaling pathway genes, and 10 transcripts were identified (Figure 7E). Among these genes, NLRP3, TNFAIP3, and TRAF6 are of particular interest, since all play a role in macrophage polarization.^[^ ^43^, ^44^, ^45^ ^]^ qPCR analysis confirmed the regulation of NLRP3 and TRAF6 by miR‐1246 in TAMs (Figure S6B, Supporting Information). As an additional control, miR‐1246 inhibitors were used to deplete the miR‐1246 variant in TAMs, and the successful inhibition of the miR‐1246 variant led to significant upregulation of NLRP3 and TRAF6 (Figure 7F and Figure S6C, Supporting Information).

NLRP3 serves as a macrophage switch (from M2 to M1 state) and its expression was highest in M1 macrophages, intermediate in M2 macrophages, and lowest in TAMs (Figure 7G and Figure S6D, Supporting Information). Moreover, both miR‐1246 mimics and variant downregulated NLRP3 in M0 macrophages and TAMs (Figure 7H), triggering an anti‐inflammatory phenotype with impaired release of TNFα and IL1β (Figure 7I and Figure S6E, Supporting Information). This process was reversed when re‐expressing NLRP3 in miR‐1246 variant‐enforced macrophages (Figure 7I,J and Figure S6F–H, Supporting Information). Further, knocking down NLRP3 in TAMs transfected with miR‐1246 inhibitors reversed the upregulation of M1 markers, but led to the upregulation of M2 marker genes (Figure 7K). TRAF6, a critical TNF receptor‐associated factor, was confirmed to be regulated by miR‐1246, but its alteration separately did not cause macrophage polarization (Figure S6I,J, Supporting Information). To understand the clinical significance of NLRP3 and TRAF6 in CRC, the transcriptomic data of READ patients were analyzed.^[^ ^46^ ^]^ The Kaplan–Meier plot demonstrated that low expression of NLRP3 and TRAF6 correlated significantly with improved patient survival (Figure S6K, Supporting Information). Taken together, our results establish that exogenous miR‐1246 partially targets NLRP3 to modulate TAM polarization.

### 2.8 miR‐1246‐Polarized Macrophages Impede the Infiltration and Functional State of CD8^+^ T Cells

**Figure 8:**
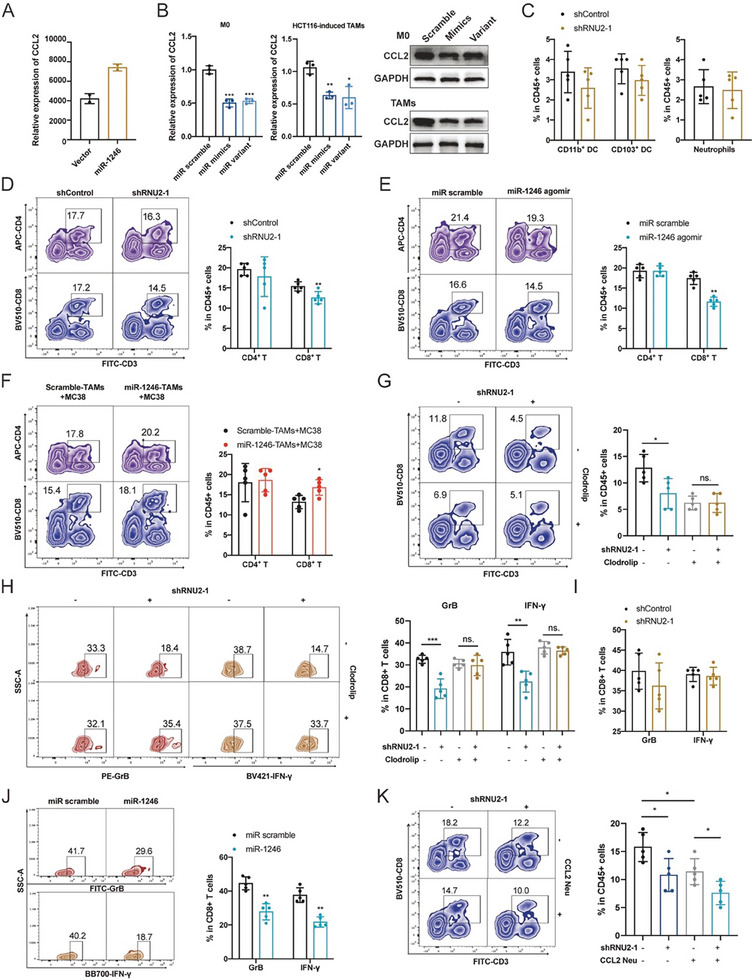
miR‐1246‐polarized macrophages impede the infiltration and functional state of CD8^+^ T cells. A) RNA‐seq data of the expression of CCL2 in TAMs transfected with miR‐1246 variant or control vector. B) The mRNA and protein levels of CCL2 in M0 macrophages and TAMs transfected with miR‐1246 mimics, variant, and miR scramble. C) Flow cytometry analysis of tumor‐infiltrating CD11b^+^ and CD103^+^ DCs in CD45^+^ cells from shControl and shRNU2‐1 mice. D) The percentage of tumor‐infiltrating CD4^+^ and CD8^+^ T cells among CD45^+^ cells from shControl and shRNU2‐1 mice. E) The percentage of tumor‐infiltrating CD4^+^ and CD8^+^ T cells among CD45^+^ cells from subcutaneous mice model bearing miR‐1246 agomir or normal control treatment. F) A mixture of miR‐1246‐induced TAMs and MC38 cells was injected subcutaneously into the flanks of C57BL/6 mice, and the proportion of tumor‐infiltrating CD4^+^ and CD8^+^ T cells was assessed. G) Flow cytometry analysis of the proportion of CD8^+^ T cells among CD45^+^ cells from shRNU2‐1 mice after Clodrolip treatment. H) The production of IFN‐γ and GrB in tumor‐infiltrating CD8^+^ T cells from shRNU2‐1 mice after Clodrolip treatment. I) The production of IFN‐γ and GrB of CD8^+^ T cells cocultured with HCT116 RNU2‐1 knockdown cells. J) The production of IFN‐γ and GrB of CD8^+^ T cells cocultured with TAMs transfected with miR‐1246 variant or miR scramble. K) The percentage of infiltrated CD8^+^ T cells in shRNU2‐1 tumors treated with or without CCL2 neutralizing antibody. Bar graphs show the mean ± SD. */**/***p < 0.05/0.01/0.001.

CD8^+^ T cells are known to be attracted by CCL2. Intriguingly, our transcriptomic data suggested an apparent upregulation of CCL2 in TAMs transfected with the miR‐1246 variant, although this increase was not statistically significant (Figures 7B and **8**A). Contrarily, our in vitro analysis indicated that the level of CCL2 was downregulated by exosomal miR‐1246 in both M0 macrophages and TAMs (Figure 8B). Given the pivotal role of CCL2 in guiding CD8^+^ T cell infiltration, we proceeded to directly evaluate the influence of exosomal miR‐1246 on CD8^+^ T cells. In a subcutaneous tumor model using shRNU2‐1 MC38 cells in C57BL/6J mice, we profiled various immune cells present in the tumor samples including neutrophils, DCs, and T cells. Flow cytometry analysis indicated that the proportion of neutrophils, DCs, and CD4**^+^** T remained unchanged between shControl and shRNU2‐1 mice (Figure 8C). However, the distribution of CD8**^+^** T cells was significantly diminished in the shRNU2‐1 mice (Figure 8D). A similar pattern emerged in subcutaneous tumors of wild‐type MC38 cells following intravenous injection with miR‐1246 agomir (Figure 8E). In another subcutaneous mice model, RAW264.7 cells were induced to TAMs with MC38 cells in vitro. The subcutaneous inoculation was performed with a mixture of miR‐1246 educated‐TAMs and MC38 cells, we observed that the recruited CD8**^+^** T cells were significantly reduced upon miR‐1246 education on week 2 (Figure 8F).

To better understand the role of miR‐1246‐induced macrophages on CD8**^+^** T cells, macrophages were depleted using Clodrolip in the shRNU2‐1 mice model. Flow cytometry analysis indicated that macrophage depletion negated the effect of shRNU2‐1 in decreasing infiltrated CD8**^+^** T cells (Figure 8G), and the cytokine production of CD8**^+^** T cells, especially IFN‐γ and GrB, were incomparable between shControl and shRNU2‐1 mice (Figure 8H). To validate these findings, human CD8^+^ T cells were isolated and cocultured with HCT116 RNU2‐1 knockdown cells. The capacity of CD8**^+^** T cells to produce IFN‐γ and GrB was determined, but no differences were observed (Figure 8I), implying that CRC cells could not alter the functional state of CD8**^+^** T cells on their own. Pursuing this, CD8**^+^** T cells were further incubated with miR‐1246/control‐induced macrophages. Unexpectedly, suppressed expression of IFN‐γ and GrB was observed when coculturing CD8**^+^** T cells with macrophages treated with miR‐1246 (Figure 8J). Furthermore, to elucidate the significance of CCL2 in the modulation of CD8^+^ T cells by miR‐1246, we employed a CCL2‐neutralizing antibody to nullify CCL2 activity in the shRNU2‐1 and control mice in vivo. Notably, the CCL2 neutralizing antibody amplified the effects observed from RNU2‐1 knockdown. However, the potential involvement of other cytokines in this regulatory cascade remains to be explored (Figure 8K). These findings suggest that miR‐1246 polarized macrophages impede the infiltration and functional state of CD8+ T cells in TIME.

## 3 Discussion

TAMs represent the most abundant fraction of the myeloid cells in solid tumors. When recruited from blood‐borne monocytic precursors to the TIME, these cells are co‐opted by cancer cells to advance tumor growth, leading to a polarization toward a pro‐tumor phenotype. This highly dynamic and varied process is the result of crosstalk between tumor cells and TAMs including a switch from pro‐inflammatory M1‐like TAMs to anti‐inflammatory M2‐like TAMs. The TIME typically abounds in M2‐like TAMs, thus creating an immunosuppressive niche for the cancer cells. Targeting specific TAM subsets and impairing their functional activation may therefore offer a novel therapeutic approach for cancer immunotherapy. However, the underlying mechanisms of this phenotypical transition in CRC are not well understood.

Macrophages are typically classified into two subtypes, namely classically activated M1 and alternatively activated M2 macrophages. Ample evidence suggests that TAMs in the TIME possess distinct characteristics that cannot fit into this traditional dichotomy. Therefore, replicating TAMs using IL‐4‐stimulated M2 macrophages in vitro, as suggested in recent studies, may not be the best choice. Instead, cancer cell‐induced macrophages bear closer similarities to TAMs in the TIME and were therefore utilized in our study. Here, we thoroughly investigated the fundamental role of cancer cells in driving macrophage polarization. Our experiments confirmed that the co‐culture of macrophages with cancer cells induced polarization toward a pro‐tumor phenotype in vitro. Moreover, traditional markers of M1 and M2 macrophages were co‐expressed in the TAMs, emphasizing the diversity of phenotype and function between these TAMs and the alternatively activated M2 macrophages.

The communication between cancer cells and TAMs is orchestrated by small molecules such as extracellular vesicles (exosomes and microvesicles), short peptides, secretory proteins, and others. Currently, considerable efforts have been devoted to exosomes, owing to their exceptional traits in favoring cell proliferation, immune responses, and metastasis of cancer cells. Unsurprisingly, as the principal RNA species encapsulated in exosomes have been shown to possess crucial oncogenic properties. In ovarian cancer, Pinar Kanlikilicer et al. demonstrated exosomes containing miR‐1246 conferred chemoresistance by regulating the polarization of M2 macrophages.^[^ ^25^ ^]^ Furthermore, in lung cancer, tumor‐initiating cell‐specific miR‐1246 promoted tumorigenicity. In CRC, the miR‐1246 serum level was elevated but reduced after primary tumor resection. Despite the lack of convincing evidence supporting the mechanisms involved, several studies showed that miR‐1246 reprogrammed macrophages into a tumor‐supporting type in CRC. In this context, our results validated the upregulation of exosomal miR1246 in CRC and reached the conclusion that miR‐1246‐enriched exosomes play an important role in the control of macrophage polarization. Our understanding is based on the following observations: first, treating macrophages with CRC‐derived exosomes resulted in the reprogramming of macrophages to M2‐like TAMs phenotypically and functionally. Second, transfecting TAMs with miR‐1246 mimics or inhibitors altered the expression levels of M2 marker genes. Third, administrating miR1246 mimics in nude mice increased the proportion of TAM in vivo. Additionally, our results established the role of miR‐1246 in the infiltration and function of CD8+ T cells, thus remodeling the immunosuppressive microenvironment to enhance antitumor immunity in the TIME.

Intriguingly, METTL3 and POU5F1 (Oct4) were reported to affect miR‐1246 levels in cancer cells. For METTL3, the mechanism involved in m6A modification was delineated; and for POU5F1, the transcriptional regulation of precursors was recognized. However, knockdown assays failed to modulate exosomal miR‐1246 levels in CRC. We noticed that the mature sequence of miR‐1246 overlapped with a region of RNU2‐1 transcript, but the commonly used stem‐loop TaqMan method struggled to differentiate between miR‐1246 and RNU2‐1‐originated variant. Thus, we introduced specifically designed primers targeting miR‐1246 and the product of RNU2‐1 degradation, revealing that exosomal miR‐1246 is mostly derived from RNU2‐1 degradation in CRC, consistent with prior findings. Moreover, our results showed that the two miR‐1246 not only shared the same sequence but also were biologically active in CRC cells. Of great significance, artificially decaying RNU2‐1 transcripts led to an increase in exosomal miR‐1246 level, which polarized macrophages into a tumor‐supporting type both in vitro and in vivo.

The functions of U2 snRNA (RNU2) require stable expression in cells, and as such, the tightly controlled decay process is imperative to eliminate the senescent or defective transcripts. This regulation is achieved through modifications at the 5′ and 3′ ends that control RNA degradation. The 3′ end uridylylation, facilitated by TUT4 and TUT7, modulates RNA decay by adding uridine to the terminal of specific RNAs. For instance, TUT4/7‐mediated uridylation of pre‐miR‐324 repositions the action of DICER and shifts the cleavage site of pre‐miR‐324, leading a strand switch from the 5′ strand (5p) to the 3′ strand (3p). In contrast, TUT1, another terminal uridylyltransferase, confers protection to U6 snRNA from exonucleases and maintains an adequate amount of U6 snRNP. Notably, the expression of TUT7 in CRC samples was found upregulated, and its impaired expression leads to a decrease in the expression of exosomal miR‐1246. Resembling the process of mRNA degradation, uridylated snRNA requires 5′–3′ or 3′–5′ exonuclease. Our examination of DIS3L2's role in generating exosomal miR‐1246 failed to yield any significant results, thereby necessitating further exploration.

Exosome sorting and secretion are stringent processes, regulated by proteins such as heterogeneous nuclear ribonucleoproteins (hnRNPs) including hnRNPA2B1 and SYNCRIP. These proteins are key players in this process, as functional assays have shown that hnRNPA2B1 recognizes the GGAG motif, while SYNCRIP recognizes the GCUG for miRNA sorting. Sequence analysis of exosomal miRNAs has revealed the overrepresentation of certain motifs, with the core motif GGAG, in these exosomes. Our results indicate that exosomal miR‐1246, derived from the RNU2‐1 fragment, harbors two GGAG motifs, with the one near the 3′ end performing the sorting role. As previously reported, the exogenous miR‐1246 variant has the capacity to reprogram macrophages and RNA‐seq analysis was employed to unravel the underlying signaling in TAMs. This analysis confirmed the involvement of cytokine interaction pathways in the regulation of macrophage polarization, with the NOD‐like receptor (NRL) signaling pathway also identified as a key mediator. Specially, NLRP3, as a core inflammasome within the NRL family, was downregulated in TAMs and found to exert a switch role in macrophage polarization, with re‐expression of this protein leading to a polarization of macrophages toward the M1 state.

**Figure 9:**
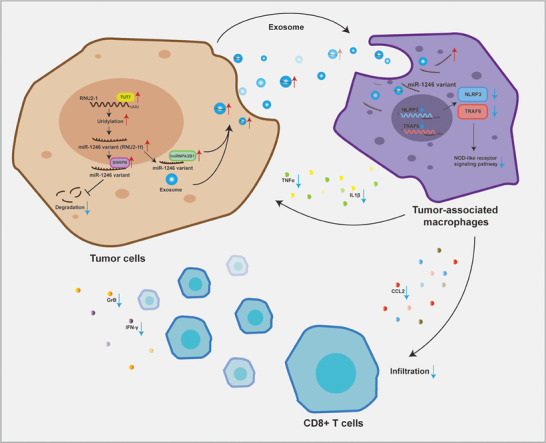
Schematic for the mechanism of exosomal miR‐1246 polarizes macrophages to support tumor progression in colorectal cancer.

In conclusion, our research highlights the crosstalk between CRC cells and macrophages in the TIME. Through the transfer of exosomal miR‐1246, tumor cells reprogram macrophages into a tumor‐supporting type (M2‐like TAMs), partially via repressing NLRP3 expression. Although the upregulation of miR‐1246 in exosomes has been noticed for years, there is limited understanding of this phenomenon. Our findings demonstrate that the upregulation of TUT7, which catalyzes RNU2‐1 uridylation for decay in CRC cells, increases exosomal miR‐1246, and this is achieved through hnRNPA2B1 sorting (**Figure** **9**). Our study has uncovered a novel axis of regulation in macrophage polarization and holds potential as a new therapeutic strategy for CRC.

## 4 Experimental Section

### Patient Samples

CRC samples were obtained from patients who underwent surgery at Jiangsu Province Hospital between January 2018 and September 2019, and all enrolled patients provided informed consent. Paired serum specimens were procured prior to and post‐tumor resection. All tumors and adjacent normal tissues were collected immediately after surgical resection and promptly transferred to a −70 °C liquid nitrogen freezer for future use. The experiments were conducted in accordance with relevant government regulations and compliance with the principles outlined in the Helsinki Declaration.

### Cell Lines and Culture

CRC cell lines (HCT116, DLD‐1), murine colon adenocarcinoma cell lines (MC38), RAW264.7), and the human monocyte cell line (THP‐1) were purchased from the Cell Bank of Type Culture Collection of the Chinese Academy of Sciences (Shanghai, China) and cultured them as specified by the supplier's instructions. HCT116 cells were cultured in McCoy's 5A medium (Wisent Bio, China) and supplemented with 10% fetal bovine serum (FBS) (Wisent Bio, China or Exosome‐Depleted FBS, Gibco, USA) and 1% penicillin/streptomycin (Thermo Fisher Scientific). DLD‐1 cells were cultured in RPMI 1640 (Wisent Bio, China) supplemented with 10% FBS and 1% penicillin/streptomycin (P/S). MC38 cells were cultured in DMEM (Wisent Bio, China) supplemented with 10% FBS and 1% P/S. THP‐1 and RAW264.7 cells were cultured in RPMI 1640 medium, supplemented with 0.05 mm 2‐mercaptoethanol (Sigma‐Aldrich), 1% P/S, and 10% FBS. The human PBMCs were isolated from buffy coats obtained from the blood of healthy donors using Ficoll density gradient centrifugation and were cultured to differentiate into macrophages. The fresh isolated PBMC were seeded on six‐well plates and cultured for 1–1.5 h in RPMI 1640 supplemented with 1% P/S. The medium and floating cells were aspirated, and a complete macrophage medium was added. The cells were then cultured for 7 days to allow them to differentiate into macrophages. All cells were incubated at 37 °C with 5% CO_2_, and mycoplasma contamination was tested routinely (Universal Mycoplasma Detection Kit, ATCC).

### Activation of Human Macrophages

To obtain activated macrophages, THP‐1 and RAW264.7 cells were treated with phorbol 12‐myristate‐12 acetate (PMA) (Abcam, 75 ng mL^−1^) for 48 h, after which the culture medium was replaced with fresh RPMI 1640 medium, supplemented with 0.05 mm 2mercaptoethanol, 1% P/S, and 10% FBS. M1 polarization was acquired by LPS (100 ng mL^−1^, Sigma) and IFNγ (20 ng mL^−1^, Peprotech) stimulation on M0 macrophages for 24 h. To obtain the M2 polarization, M0 macrophages were treated with IL4 (20 ng mL^−1^, RND) and IL14 (20 ng mL^−1^, Miltenyi) for 24 h.

### Induction of Tumor‐Associated Macrophages In Vitro

To educate TAMs in vitro to better mimic the complexity of TAMs in CRC, the macrophages were cocultured with CRC cells. Briefly, 4 × 10^5^ DLD‐1 and HCT116 cells were seeded in a Transwell insert (24 mm diameter, 0.4‐µm pore size, CORNING), and cultured with cancer medium in a six‐well plate for 36 h, until a desired cell density was attained. Subsequently, the cancer cell medium was replaced with a macrophage medium (fresh RPMI 1640 medium, supplemented with 0.05 mm 2‐mercaptoethanol, 1% P/S, and 10% FBS) and incubated for an additional 24 h. The Transwell insert containing cancer cells was then transferred to a new six‐well plate, in which M0 macrophages were cultured, and incubated for 5 days.

### RNA Extraction and qRT‐PCR Analysis

Total RNA was extracted from cancer cells and tumor‐derived exosomes (10 µg in 20 mL of PBS) using the Invitrogen TRIzol reagent (Thermo Fisher Scientific, USA), and quantified using the NanoDrop 2000 (Thermo Fisher Scientific). The extracted RNA was then reverse‐transcribed into complementary DNA (cDNA) using the PrimeScript RT‐PCR Kit (TaKara) according to the manufacturer's instructions. For miRNA profiling, the TaqMan MicroRNA Reverse Transcription Kit (Thermo Fisher Scientific) and TaqMan Universal PCR Master Mix (Thermo Fisher Scientific) were used. For the poly‐A tailing SYBR qRT‐PCR method, cDNA was synthesized using the MystiCqTM microRNA cDNA Synthesis Mix (Sigma‐Aldrich). The forward primer for miR‐1246 was designed and synthesized by Gene Pharma (China). The qRT‐PCR was performed using the microRNA SYBR Green qPCR ReadyMix and Universal PCR Primer (Sigma‐Aldrich), according to the manufacturer's guidelines. Both qRT‐PCR strategies were conducted on an Applied Biosystems 7500 Sequence Detection System (Applied Biosystem). The relative expression of target genes was determined through the ΔΔCT method, with mRNA level normalized to the housekeeping gene GAPDH, and miRNA levels normalized to U6. The sequences of all PCR primers are listed in Table S5, Supporting Information.

### Flow Cytometry

The human and mouse tumor samples were first dispersed into single‐cell suspensions and then incubated with the appropriate antibodies for 30 min on ice in the dark, after which the samples were subjected to FlowJo V10.4 (Beckman Coulter, USA). The antibodies used were as follows: APC Anti‐Human CD206 (550 889, BD Pharmingen), PE Anti‐Human CD86 (560 957, BD Pharmingen), PE Anti‐Mouse CD11b (101 207, BioLegend), FITC Anti‐Mouse F4/80 (123 107, BioLegend), APC‐Cy7 Anti‐Mouse CD45 (557 659, BD Pharmingen), FITC Anti‐Mouse CD3 (553 061, BD Pharmingen), APC Anti‐Mouse CD4 (561 091, BD Pharmingen), BV510 Anti‐Mouse CD8a (563 068, BD Pharmingen), PE Anti‐Mouse Granzyme B (12‐8898‐82, BD Pharmingen), BV421 Anti‐Mouse IFN‐γ (563 376, BioLegend), APC Anti‐Human CD45 (555 485, BD Pharmingen), PE Anti‐Human CD3 (555 333, BD Pharmingen), BV421 Anti‐Human CD8 (562 428, BD Pharmingen), FITC Anti‐Human CD4 (555 346, BD Pharmingen), FITC Anti‐Human Granzyme B (561 998, BD Pharmingen), BB700 Anti‐Human IFN‐γ (566 395, BD Pharmingen), PE Anti‐Mouse Ly‐6C (560 592, BD Biosciences), APC Anti‐Mouse Ly‐6G (560 599, BD Biosciences), APC Anti‐Mouse CD103 (566 722, BD Biosciences), FITC Anti‐human CD14 (130‐080‐701, Miltenyi Biotec). To characterize the composition of TIME, CD4^+^ T cells, CD8^+^ T cells, CD103^+^CD11b^+^ dendritic cells, Ly6G^+^CD11b^+^ neutrophils, and CD11b^+^F4/80^+^ macrophages were analyzed by flow cytometry.

### Cell Proliferation and Migration Assays

For proliferation analysis, 1 × 10^4^ tumor cells per well were seeded in a 96‐well plate for 24 h. The culture medium was then discarded and replaced with serum‐free medium for an additional 24 h. Then, the cancer cells were treated with a different CM for 24 h. The proliferation rate was determined using the CCK‐8 kit, following the manufacturer's instructions (Dojindo, Japan). For migration assays, tumor cells were harvested post‐treatment with different CM and plated on a Millicell cell culture insert (24‐well insert, 8‐µm pore size, Sigma‐Aldrich). Briefly, 4 × 10^4^ cells per well were suspended in 200 µL of serum‐free medium and seeded into the bottom of the inserts, and 500 uL of culture medium supplemented with 10% FBS were added to the plate. After incubation for 24 h, the inserts were rinsed with PBS, fixed with methanol, and stained with crystal violet (Beyotime Biotechnology). Three fields were randomly selected in the chambers and evaluated under a microscope to determine the number of migratory cells.

### Western Blot

The cells were lysed in cell lysis buffer (Beyotime Biotechnology), supplemented with a cocktail of protease and phosphatase inhibitors (Sigma‐Aldrich). The lysate was subjected to vigorous vortexing and then centrifuged at a speed of 120 000 × *g* for 5 min at 4 °C. The supernatant was discarded, and the precipitate was harvested. The protein concentration was measured by the Quick Start Bradford Protein Assay Kit (Bio‐Rad). For the purpose of relative quantification of the target proteins, equal amounts of protein were subjected to SDS‐PAGE electrophoresis and transferred to the PVDF membranes (Bio‐Rad). The antibodies used are listed in Table S6, Supporting Information.

### ELISA

The human IL‐1 beta and TNF alpha ELISA Kits (Abcam) were performed according to the manufacturer's instructions. Briefly, 50 µL of samples were added to the wells, followed by the addition of 50 µL of antibody cocktail in each well. The mixture was then incubated for 2 h at room temperature (RT) on a shaker set to 400 rpm. After washing the wells three times with 1× Wash Buffer PT, 100 µL of TMB development solution was added and incubated for 10 min at RT on a shaker set to 400 rpm. Finally, the reaction was halted with the Stop Solution and absorbance was measured at 450 nm using a microplate reader (Bio‐Rad, USA).

### Exosome Isolation and Characterization

To isolate exosomes, an ultracentrifugation protocol was implemented. Briefly, CRC cells were cultured for certain days, and the supernatant was collected. The medium was first centrifuged at 500 × *g* for 5 min and then centrifuged at 15 000 × *g* for 30 min to remove cellular debris. The pellets were ultracentrifuged at 110 000 × *g* for 70 min to pellet the exosomes using a centrifuge (L‐80XP; Beckman Coulter, Brea, CA, USA). To deplete contaminating proteins, the pellets were washed with 20 mL PBS and then ultracentrifuged at 110 000 × *g* for 70 min again. Subsequently, the exosome pellets were resuspended in nuclease‐free water, followed by exosome characterization, RNA/protein extraction, and other uses. All the procedures were performed at 4 °C. The diameter and distribution of extracted exosomes were determined by NTA using a ZetaView instrument (Particle Metrix) following the manufacturer's instructions. The morphology of purified exosomes was analyzed by TEM.

### Mouse Model

Mice were obtained from the animal center of Nanjing Medical University (Nanjing, China) and were maintained under pathogen‐free conditions. All animal studies were performed in accordance with the experimental animal use guideline of the National Institutes of Health and were approved by the Institutional Animal Care and Research Advisory Committee of Nanjing Medical University. To determine the effects of miR‐1246‐educated macrophages on tumor growth, TAMs were first co‐cultured with miR1246‐mimics for 24 h, and then incubated (2 × 10^5^) with 1 × 10^6^ CRC cells for another 24 h. For the subcutaneous tumor model, BALB/c nude mice or C57BL/6J aged 6 weeks were inoculated subcutaneously with the indicated cells, and the tumor growth was monitored at certain time points. Tumor volumes were calculated using the formula *a* × *b* ^2^/ 2 (*a*, longest diameter; *b*, shortest diameter). After 4 weeks, the mice were sacrificed, and the tumor samples were excised for weighing macrophage isolation, or flow cytometry. Part of the tumors was fixed and embedded in paraffin, and subjected to Immunohistochemistry (IHC) staining. For the metastatic model, 6‐week‐old BALB/c nude mice were used. After the mice were anesthetized, 1 × 10^6^ mixed cells resuspended in 20 µL PBS were injected into the distal tip of the spleen using a 29G needle. 5 weeks later, all mice were sacrificed and livers were dissected for gross assessment, IHC, or macrophage isolation. To investigate the functions of miR‐1246 agomir in vivo, a xenograft model was first established by inoculating with CRC cells (transfected with the GFP‐luciferase vector) subcutaneously and allowing tumor growth for 2 weeks. Later, 35 mg/20 g miR‐1246 agomir and agomir NC were injected by the tail vein intravenously (iv) every 3 days. On day 0 and day 21, the mice were imaged using the IVIS imaging system. To visualize the signals of tumor samples, d‐luciferin potassium salt (100 µL of 10 mg mL^−1^ dissolved in PBS) was injected IP, and the images were taken after 10 min. Bioluminescence signals were analyzed by the Living‐Image software.

### Macrophage Depletion

C57BL/6J mice at 6 to 8 weeks of age were purchased from the animal center of Nanjing Medical University and maintained under appropriate conditions. MC38 cells (1 × 10^6^) were injected subcutaneously into the flanks of mice and allowed to grow into tumors.

After 1 week of inoculation (when tumors were palpable), 100 µL of clodronate liposome (Clodrolip) (Sigma‐Aldrich) and PBS liposome were administered intraperitoneally every 3 days until the end of the experiment.

### Macrophage Isolation

To isolate macrophages from human and mouse tumors, a tumor dissociation kit was employed (Miltenyi Biotec, Germany), in accordance with the instructions. Specifically, fibrous, fat, and necrotic tissues in the samples were first removed, and the remaining tissues were diced into 2–4 mm fragments. The prepared digestive enzyme mix was added to the samples, which were then loaded onto the gentleMACS Dissociator (Miltenyi Biotec), and the default program was performed. Subsequently, the products in this step were applied to a 70 µm strainer, followed by a 30 µm strainer, to eliminate debris. Finally, the cell suspensions were washed, centrifuged, and resuspended in an ice‐cold PBS buffer for downstream applications. To isolate TAMs from the single‐cell suspensions, magnetic activated cell sorting (MACS, Miltenyi) was performed utilizing a human CD68‐PE primary antibody (F4/80‐PE for mice), and an anti‐PE MicroBeads secondary antibody (Miltenyi Biotec) according to the manufacturer's instructions.

### Peripheral Blood Mononuclear Cell Isolation

For isolation of the PBMCs, MACSprep PBMC Isolation Kit (Miltenyi Biotec) was used according to the manufacturer's instructions. Specifically, 8 mL of human whole blood was obtained from healthy donors and added to the prepared reaction system.

The PBMCs were then harvested from the supernatant that had passed through the LS column. The magnetically sorted cells were subsequently subjected to further use. For macrophage induction, human CD14^+^ cells were first isolated using MACS (130‐050‐201, Miltenyi Biotec), and the purity of monocytes was determined by flow cytometry. Subsequently, tumor‐derived CM was utilized to induce macrophage differentiation and polarization in vitro. For CD8^+^ T cells isolation from PBMCs, the CD8 MicroBeads (130‐045‐201, Miltenyi Biotec) were used.

### Hematoxylin and Eosin and Immunohistochemistry Staining

First, the samples were embedded in paraffin and sectioned into 4 µm slices. For hematoxylin and eosin (H&E) staining (Abcam), the sections were deparaffinized and sequentially treated with H&E stains. For IHC analysis, the sections were blocked with 5% normal goat serum (Thermo Fisher Scientific) in PBS for 1 h at RT. The sections were incubated with primary antibodies overnight at 4 °C and subjected to secondary anti‐rabbit immunoglobulin G (IgG) (Abcam) incubation. Detection was performed using a digital microscope camera. The staining intensity (*i*) was quantified using the Image J software. In order to determine the CD86 or CD206 expression, the histochemistry score (H‐score), which factors in both staining intensity and the percentage of positively stained cells, was utilized. Specifically: Staining intensity (*i*) was categorized as: weak (0), medium (1), strong (2), and strongest (3). The proportion of positively stained cells (*pi*) was graded into the following ranges: ≈0–5%, ≈6–25%, ≈26–50%, ≈51–75%, and ≈76–100%. The H‐score was then computed using the formula: H‐score  =  ∑ (*pi*  ×  *i*).

### miRNA Mimics and Inhibitors

To modulate the levels of miRNA in cells, miRNA mimics, inhibitors, and scrambled controls were designed and synthesized (Gene Pharma, China). For the transfection procedures, the CRC cells or macrophages were seeded in a 6‐well plate to reach 60–80% confluence. The oligonucleotides of mimics and inhibitors were added to the cells at a final concentration of 50 nm using Lipofectamine RNAiMAX Reagent (Invitrogen). Then, the transfected cells were analyzed using qPCR. The sequences for miR‐1246 mimics, inhibitors, and scramble control are listed in Table S7, Supporting Information.

### Target Prediction of miR‐1246

TargetScan (www.targetscan.org/vert_72) and KEGG (www.genome.jp/kegg/) were used to predict the biological targets of miR‐1246. Predictions that contained at least one conserved site that matched the seed region of miR‐1246 in 3′ UTR were considered.

### RNA Knockdown with siRNA

The siRNAs were designed and synthesized by Gene Pharma (Shanghai, China). The transfection of siRNAs used the highly efficient Lipofectamine 3000 reagent (Invitrogen). Following the manufacturer's instructions, cells were cultured in 6‐well plates and transfected with siRNA at a final concentration of 50 nm for 6 h in a serum‐free culture medium. The cells were then cultured in a serum‐containing medium for another 24 h, after which knockdown efficiency was assessed by qRT‐PCR or western blot. For transfection in macrophages, the highly specialized jetPEI‐Macrophage kit (Illkirch‐Graffenstaden, France) was used. Briefly, the siRNA and transfection reagent were first pre‐mixed in an EP tube for 30 min at RT, after which the mixture was added to the serum‐containing medium of macrophages to allow transfection. After 24 h of incubation at 37 °C, the knockdown efficiency was assessed. For lentiviral shRNA transfection, shRNU2‐1 was synthesized by Gene Pharma. The cells were transfected with lentiviral shRNA in the presence of polybrene (4 mg mL^−1^) (Sigma‐Aldrich, USA). The siRNA and shRNA sequences are listed in Table S7, Supporting Information.

### Generation of RNU2‐1 Fragments (RNU2‐1) Mutations

The sequence of human RNU2‐1 was retrieved from the Ensembl database (Table S8, Supporting Information). To produce mutations of RNU2‐1f, specific sequences were modified by replacing the indicated nucleotides and synthesized by Generay (Shanghai, China).

### RNA‐seq and Enrichment Analysis

RNA‐seq analysis was performed on TAMs transfected with the miR‐1246 variant and normal control. Library preparation and sequencing followed previously established procedures. Briefly, 1 µg of total RNA was used for the library construction, using the TruSeq RNA Sample Prep Kit v2 (Illumina). First, poly‐T oligo‐at magnetic beads were used to purify mRNA from the total RNA, which was then fragmented using divalent cations in Vazyme Frag/Prime Buffer (Vazyme Biotech Co.). Next, the first strand cDNA was generated using random primers and reverse transcriptase, followed by synthesis of the second‐strand cDNA using proper buffer, DNA polymerase I, dNTPs, and RNase H. Then, a preadenylated DNA adapter was ligated to the 3′‐end of each cDNA fragment, followed by ligation with special sequencing adapters. All products were purified and size‐selected to obtain the appropriate size for sequencing. PCR was performed, and the size distribution was determined using Bioanalyzer DNA High Sensitivity Kit (Agilent Technologies) before loading into the Illumina X‐Ten for 2 × 150 bp pair‐end (PE) sequencing. All raw data were quality‐checked using FasQC (www.bioinformatics.babraham.ac.uk/projects/fastqc). The clean reads were mapped to regions in the reference genome (GRCh38.27), and gene expression analysis was performed. The DEGs were identified using the DESeq2 package, with significant differences defined by an FDR < 0.05. Up‐ or down‐regulated DEGs were subjected to enrichment analysis with the KOBAS tool (http://kobas.cbi.pku.edu.cn/genelist), which annotated the KEGG pathway and GO terms.

### miRNA Pull‐Down Assay

Biotin‐labeled miRNAs of interest were chemically synthesized at the 3′ end (Gene Pharma). For the pull‐down assay, Dynabeads M‐280 Streptavidin (Invitrogen) was used. To prepare the beads for the assay, streptavidin beads (0.5 mg) were resuspended and washed in the washing buffer for 5 min on a roller. To remove the potential RNase contamination, the beads were washed twice with Solution A for 2 min and once with Solution B, after which they were resuspended in Solution B. Then, the washed beads were mixed with 50 pmol of labeled miRNA and incubated for 30 min at RT with gentle rotation. The coated beads were separated using a magnet (Invitrogen, Life Technologies) and resuspended in 2× B&W Buffer to a final concentration of 5 µg µL^−1^. To further immobilize the beads, the 2× B&W Buffer was diluted with an equal volume of distilled water and incubated for 10 min at RT with gentle rotation. The coated beads were separated, washed, and resuspended. The biotinylated miRNA‐coated beads were then incubated with protein samples for 30 min at RT with gentle rotation, followed by washing twice with 1× binding buffer to remove unbound proteins. The protein/miRNA complex was then eluted by using the SDS‐PAGE loading buffer (50 mm Tris‐HCl, pH 7.5 + 0.1% SDS) at 85 °C for 5 min. Eluted proteins were subjected to western blot analysis.

### RNA‐Binding Protein Immunoprecipitation (RIP)

RIP was performed using the Magna RIP RNA‐Binding Protein Immunoprecipitation Kit (Millipore, USA) according to the manufacturer's instructions. Briefly, cancer cells were scraped and resuspended in a complete RIP lysis buffer with gentle pipetting. Then, the lysates were stored at −80 °C for further use. Subsequently, the magnetic beads were washed on a magnetic separator with RIP wash buffer multiple times, then 5 µg of hnRNPA2B1 antibody (Abcam) or anti‐IgG antibody (Abcam) was added and allowed to incubate with rotation for 30 min at RT. Afterward, the cell lysates were thawed, and centrifuged at 4 °C for 10 min (14 000 rpm), and the supernatant (100 µL) was transferred and incubated with the bead‐antibody complex with rotation for 24 h at 4 °C, while another 10 µL of the supernatant were labeled as “input.” After overnight incubation, the beads were collected using the magnetic separator. Then, the protein‐conjugated beads were incubated with proteinase K buffer for 30 min at 55 °C with shaking, followed by successive miRNA isolation. The reverse transcription and fold‐change analysis of miRNAs were conducted, as described earlier. The enrichment of miRNA was indicated as the percent of input.

### Statistical Analysis

The data presented in the figures are shown as mean ± standard deviation (SD). The statistical analyses were conducted using SPSS 13.0 software (Chicago, IL, USA) and GraphPad Prism software (La Jolla, CA, USA). Two‐tailed Student's *t*‐tests were used to compare means between two groups, while a one‐way analysis of variance was employed for comparisons among more than two groups. Statistical significance was defined as *p* < 0.05.

## Conflict of Interest

The authors declare no conflict of interest.

## Supporting information

Supporting Information

## Data Availability

The data that support the findings of this study are available from the corresponding author upon reasonable request.
