# Supplementary material for: The Uridylyl Transferase TUT7‐Mediated Accumulation of Exosomal miR‐1246 Reprograms TAMs to Support CRC Progression
Source: Adv Sci (Weinh). 2024 Feb 11;11(15):2304222. doi: 10.1002/advs.202304222 (PMC11022710; doi:10.1002/advs.202304222)
Supplement: Supplementary file 1 — Supporting Information [file ADVS-11-2304222-s001.pdf]

ADVANCED SCIENCE
Open Access

Supporting Information

for *Adv. Sci.*, DOI 10.1002/advs.202304222

The Uridylyl Transferase TUT7-Mediated Accumulation of Exosomal miR-1246 Reprograms TAMs to Support CRC Progression

Yifei Feng, Chi Jin, Tuo Wang, Zhihao Chen, Dongjian Ji, Yue Zhang, Chuan Zhang, Dongsheng Zhang, Wen Peng and Yueming Sun**

Supporting Information for

The Uridylyl Transferase TUT7-Mediated Accumulation of Exosomal miR-1246 Reprograms TAMs to Support CRC Progression

This file includes:

Supplementary Figures: S1 to S6

Supplementary Tables: S1 to S9

Fig.S1

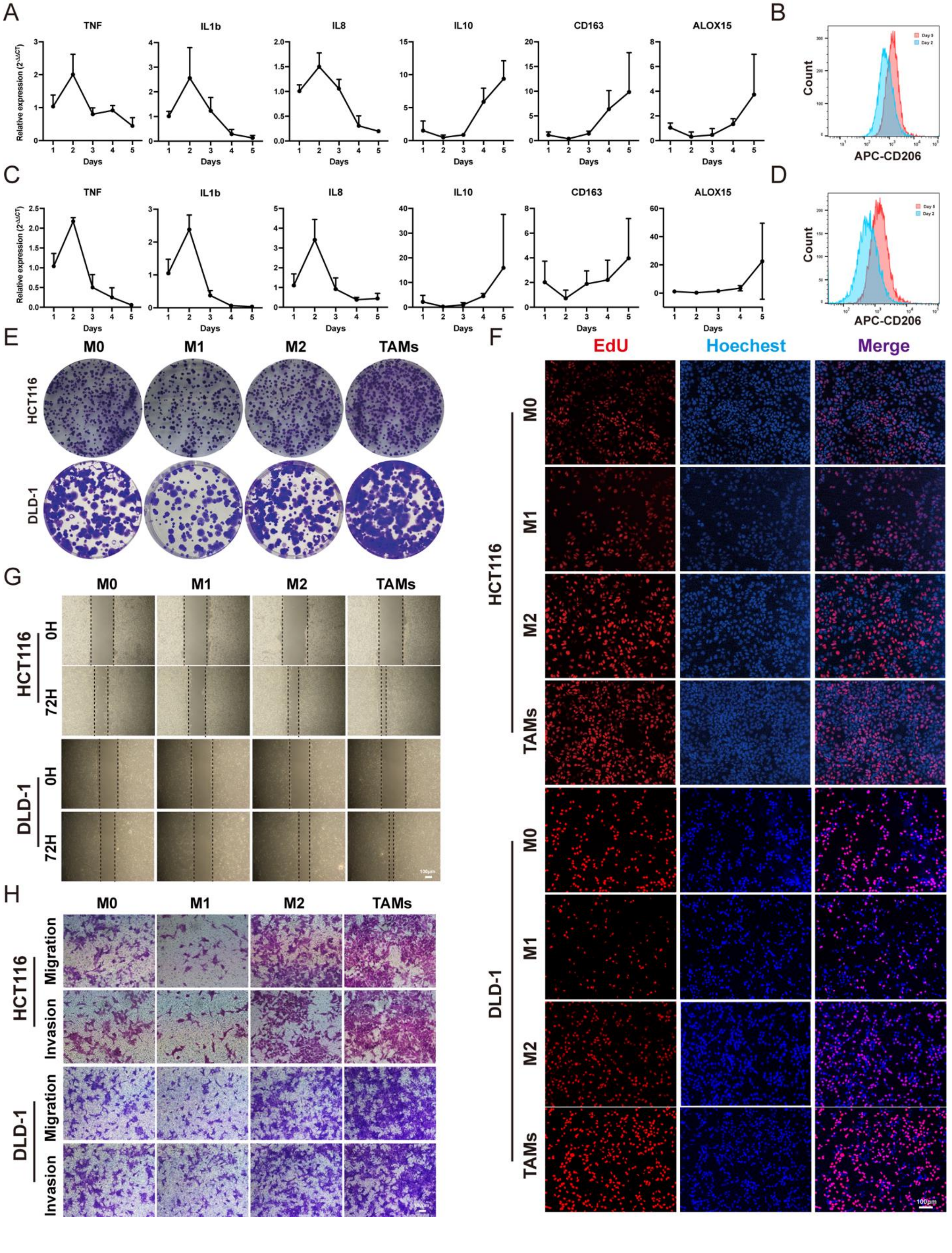
A
TNF
IL1b
IL8
IL10
CD163
ALOX15
Relative expression (2^-ΔΔCT)
Days
B
Count
APC-CD206
Day 5
Day 2
C
TNF
IL1b
IL8
IL10
CD163
ALOX15
Relative expression (2^-ΔΔCT)
Days
D
Count
APC-CD206
E
M0
M1
M2
TAMs
HCT116
DLD-1
F
EdU
Hoechest
Merge
HCT116
DLD-1
M0
M1
M2
TAMs
100μm
G
M0
M1
M2
TAMs
HCT116
DLD-1
0H
72H
100μm
H
M0
M1
M2
TAMs
HCT116
DLD-1
Migration
Invasion

Fig.S1 Cancer Cells Reprogram Macrophages to A Tumor-supporting Type. **A and C**, The mRNA expression of macrophage markers (TNF, IL1b, and IL8 for M1 type; IL10, CD163, and ALOX15 for M2 type) in a coculture system with PBMC-derived macrophages and DLD-1 cells (upper panel) /HCT116 (lower panel) cells at each time point. **B and D**, Flow cytometry of CD206 in macrophages induced by cancer cells from the particular time point, day 5 versus day 2. **E**, Colony formation of cancer cells cocultured with conditioned medium (CM) from M0, M1, M2, and TAMs. Representative images are shown. **F**, EdU assay of HCT116 and DLD-1 cells cocultured with indicated macrophages was performed, and representative images are shown. Bars = 100 μm. **G**, Wound healing assays of HCT116 and DLD-1 cells cocultured with indicated macrophages were performed, and representative images are shown. Scale bars = 100 μm. **H**, The migration and invasion assays of HCT116 and DLD-1 cells cocultured with indicated macrophages were performed and representative images are shown. Scale bars = 100 μm. Bar graphs represent the mean + SD. */**/***/****P< 0.05/0.01/0.001/0.0001.

Fig.S2

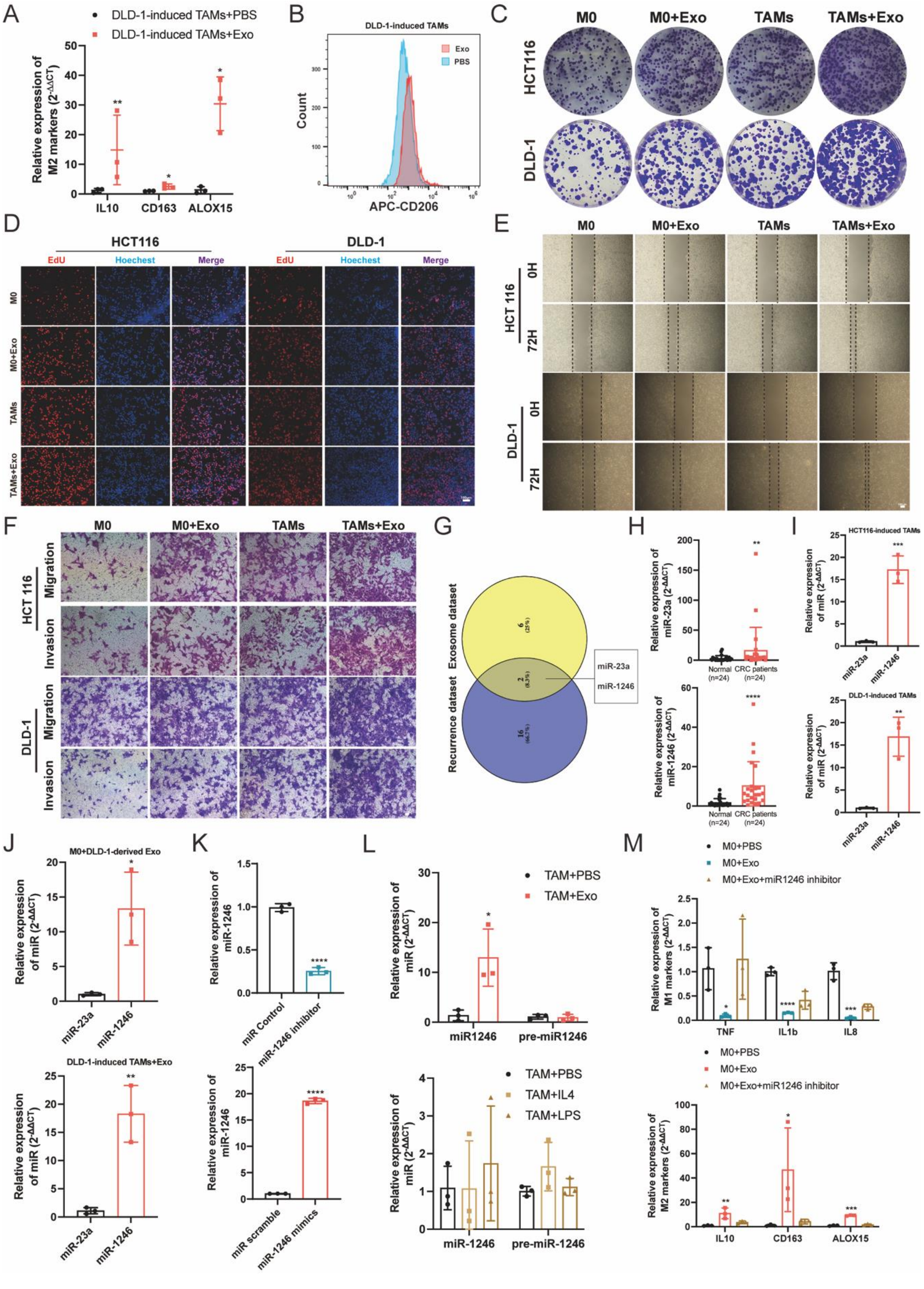
A
DLD-1-induced TAMs+PBS
DLD-1-induced TAMs+Exo
Relative expression of M2 markers (2^-ΔΔCT)
IL10
CD163
ALOX15
B
DLD-1-induced TAMs
Exo
PBS
Count
APC-CD206
C
M0
M0+Exo
TAMs
TAMs+Exo
HCT116
DLD-1
D
HCT116
DLD-1
EdU
Hoechest
Merge
M0
M0+Exo
TAMs
TAMs+Exo
E
M0
M0+Exo
TAMs
TAMs+Exo
HCT 116
0H
72H
DLD-1
0H
72H
F
M0
M0+Exo
TAMs
TAMs+Exo
HCT 116
Migration
Invasion
DLD-1
Migration
Invasion
G
Exosome dataset
Recurrence dataset
miR-23a
miR-1246
H
Relative expression of miR-23a (2^-ΔΔCT)
Relative expression of miR-1246 (2^-ΔΔCT)
Normal (n=24)
CRC patients (n=24)
I
HCT116-induced TAMs
DLD-1-induced TAMs
Relative expression of miR (2^-ΔΔCT)
miR-23a
miR-1246
J
M0+DLD-1-derived Exo
DLD-1-induced TAMs+Exo
Relative expression of miR (2^-ΔΔCT)
miR-23a
miR-1246
K
Relative expression of miR-1246
miR Control
miR-1246 inhibitor
miR scramble
miR-1246 mimics
L
TAM+PBS
TAM+Exo
Relative expression of miR (2^-ΔΔCT)
miR1246
pre-miR1246
TAM+PBS
TAM+IL4
TAM+LPS
miR-1246
pre-miR-1246
M
M0+PBS
M0+Exo
M0+Exo+miR1246 inhibitor
Relative expression of M1 markers (2^-ΔΔCT)
TNF
IL1b
IL8
M0+PBS
M0+Exo
M0+Exo+miR1246 inhibitor
Relative expression of M2 markers (2^-ΔΔCT)
IL10
CD163
ALOX15

Fig.S2 Polarization of Macrophages by Exosomal miR-1246. **A**, The mRNA level of M2 marker genes in M0 and DLD-1-induced TAMs, treated with cancer-cell derived exosomes or PBS. **B**, Flow cytometry analysis demonstrated the fluorescence intensity of CD206 positive macrophages after treating with DLD-1-derived exosomes or PBS control. **C to F**, Representative images of proliferation and metastasis assays with the indicated treatment. Scale bars = 100 μm. Biological replicates are in triplicate (n=3). **G**, Overlapping the two datasets identified two candidate miRNA, miR-23a and miR-1246. **H**, qPCR analysis of miR-23a and miR-1246 in serum exosomes from CRC patients and healthy donors. **I**, qPCR analysis of miR-23a and miR-1246 in DLD-1/HCT116-induced TAMs. **J**, qPCR analysis of miR-1246 level in M0 macrophages (upper panel) and DLD-1-induced TAMs (lower panel), which were pretreated with DLD-1-derived exosomes. **K**, The knockwdown or overexpression efficancy of miR-1246 in macrophages using miRNA mimics or inhibitors. **L**, The expression of miR-1246 and pre-miR-1246 in TAMs (DLD-1 induced) in the presence of exosomes, IL4, LPS or PBS. **M**, M0 macrophages cultured with DLD-1-derived exosomes, followed by transfection with miR-1246 inhibitors, and then the expression of M1 and M2 marker genes was assessed by qPCR. Bar graphs show mean + SD. */**/***P< 0.05/0.01/0.001. Bar graphs show mean + SD. */**/***/****P< 0.05/0.01/0.001/0.0001.

Fig.S3

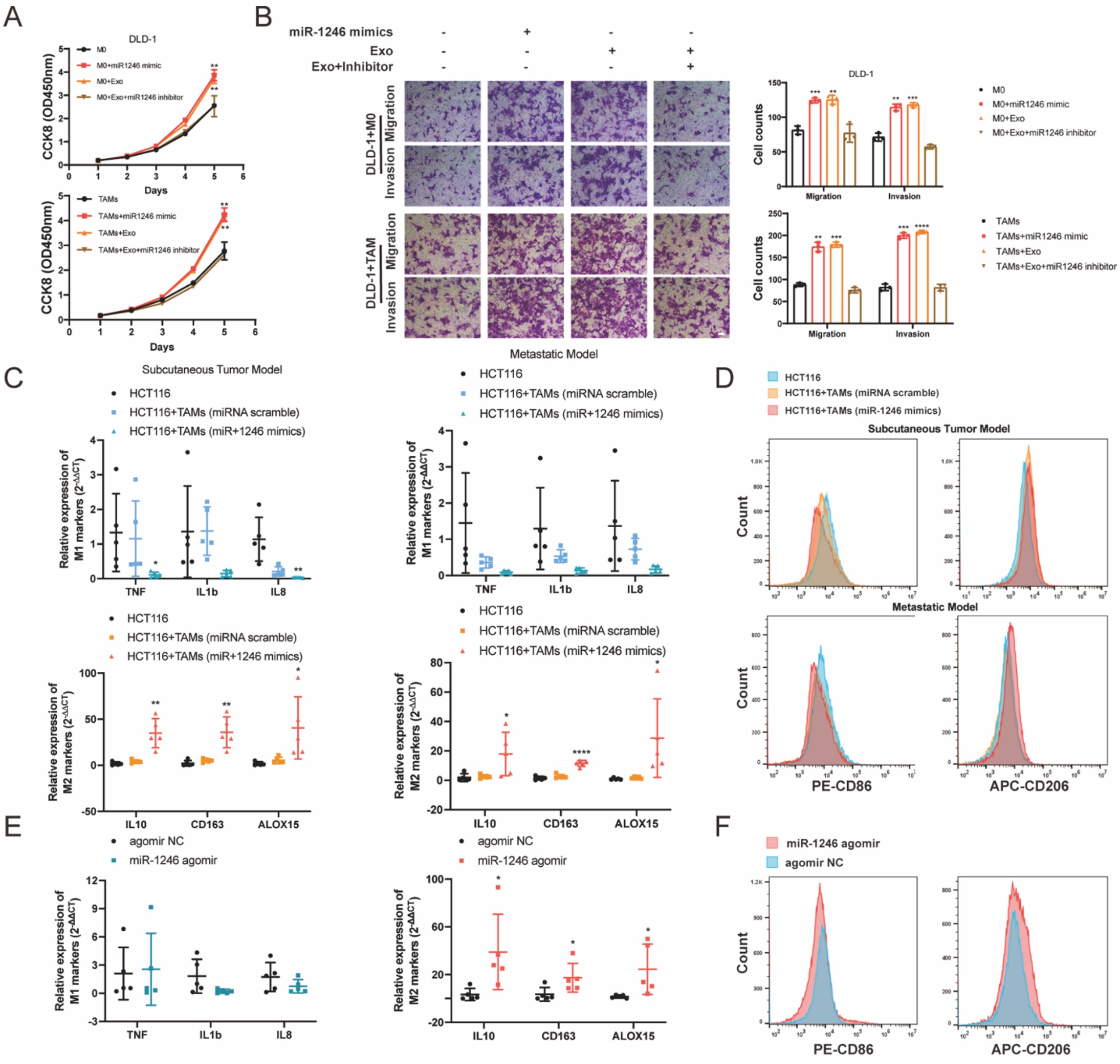

Fig.S3 miR-1246-Induced Macrophages Promote CRC Progression. **A and B**, CCK-8 and transwell analysis of DLD-1 cells in the presence of CM from M0 macrophages or TAMs transfected with the miR-1246 mimics, miR-1246 inhibitors, or exosomes for 24 hours. Scale bars = 100 μm. **C and D**, The macrophages extracted from subcutaneous and metastatic tumor models were subjected to qPCR for the detection of M1/M2 marker genes and to flow cytometry for CD86/CD206 analysis. **E**, qPCR analysis of marker genes of macrophages extracted from subcutaneous mice model after treatment of miR-1246 agomir. **F**, Flow cytometry of CD86 and

CD206 in macrophages isolated from the subcutaneous mice model after treatment of miR-1246 agomir. Bar graphs show mean + SD. */**/***/****P< 0.05/0.01/0.001/0.0001.

Fig.S4

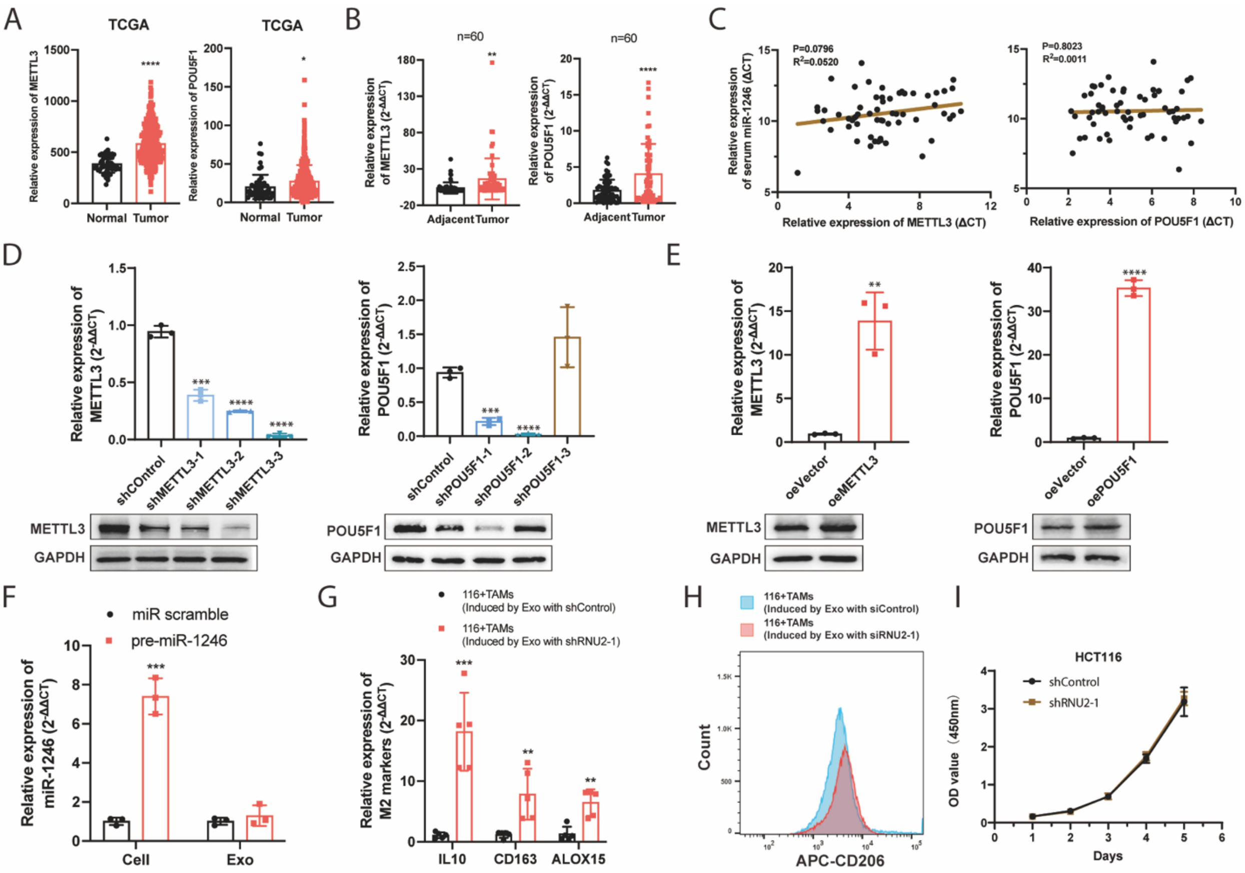

Fig.S4 RNU2-1-derived Exosomal miR-1246 Contributes to Macrophage Polarization. **A**, The expression patterns of METTL3 and POU5F1 in TCGA COADREAD cohort. **B**, qPCR analysis of METTL3 and POU5F1 levels in tumor samples of CRC patients, n = 60. **C**, The Pearson correlation analysis of the expression between METTL3 in tumor samples and serum miR-1246, and between POU5F1 in tumor samples and serum miR-1246. **D**, The knockdown efficiency of METTL3 and POU5F1 in HCT116 cells, assessed by qPCR analysis and western blot. **E**, The overexpression efficiency of METTL3 and POU5F1 in HCT116 cells, assessed by qPCR analysis and western blot. **F**, The levels of cellular miR-1246 and exosomal miR-1246 in HCT116 cells with pre-miR-1246 transfection. **G and H**, The polarized-macrophages by indicated exosomes were mixed with HCT116 cells and were then to form xenograft tumor model. Macrophages extracted from the above were subjected to qPCR and flow cytometry analysis. **I**, CCK-8 analysis of MC38 cells transfected with shRNU2-1 and normal control. Bar graphs show mean + SD. */**/***/****P< 0.05/0.01/0.001/0.0001.

Fig. S5

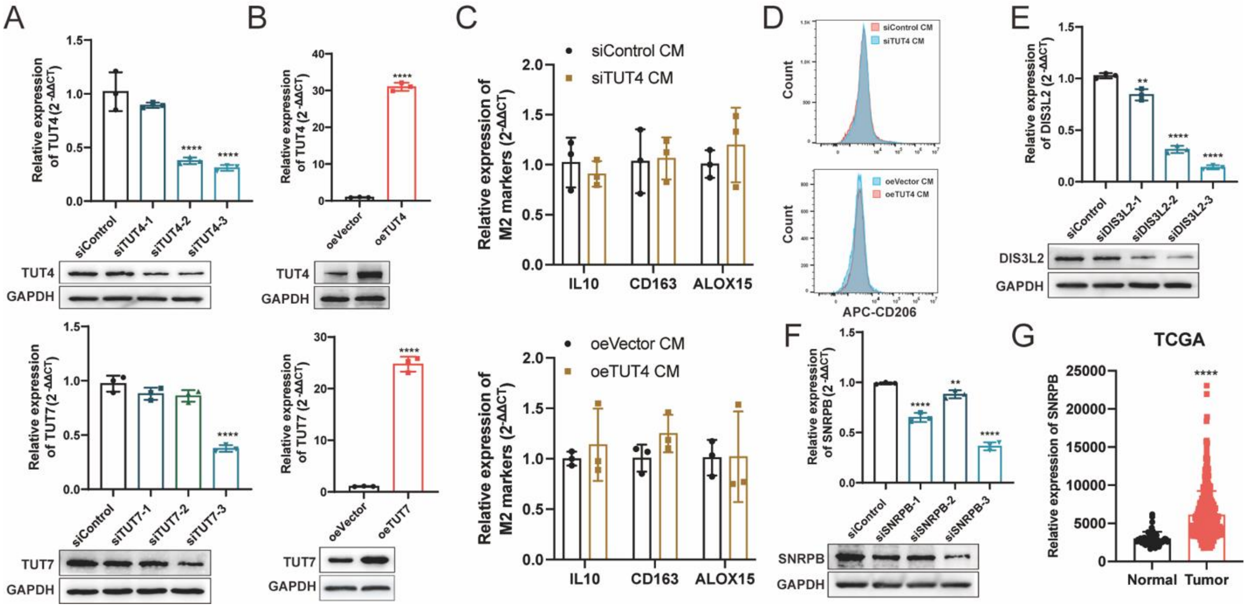

Fig. S5 TUT7 Facilitates the Accumulation of Exosomal miR-1246 in CRC. **A**, The knockdown efficiency of TUT4 (upper panel) and TUT7 (lower panel) in HCT116 cells, assessed by qPCR and western blot. **B**, The overexpression efficiency of TUT4 (upper panel) and TUT7 (lower panel) in HCT116 cells, assessed by qPCR and western blot. **C**, The expression of M2 marker genes in macrophages, induced by CM from TUT4 knockdown or overexpression cancer cells. **D**, Flow cytometry of CD206 in macrophages induced by CM from TUT4 knockdown or overexpression cancer cells. **E and F**, The knockdown efficiency of DIS3L2 and SNRPB in HCT116 cells, assessed by qPCR analysis and western blot. **G**, SNRBP level from TCGA data. Bar graphs show mean + SD. **/***/****P< 0.01/0.001/0.0001.

Fig.S6

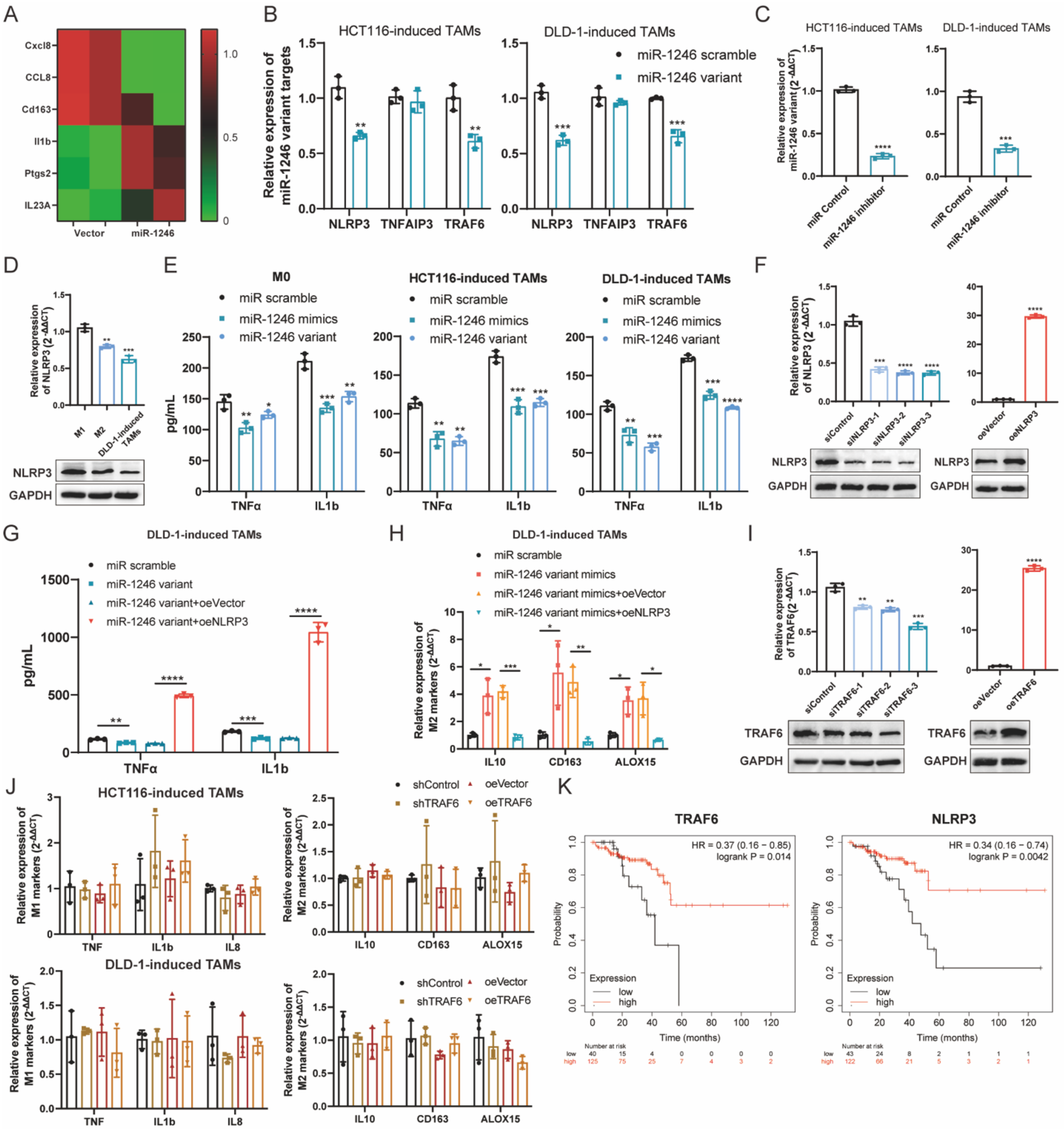

Fig.S6 Exogenous miR-1246 Targets NOD-Like Receptor Pathway to Regulate Polarization of TAMs. **A**, The heatmap of M1 and M2 marker genes in TAMs transfected with miR-1246 variant or vector control. **B**, Relative expression of NLRP3, TNFAIP3, and TRAF6 in TAMs transfected with miR1246 variant or normal control. **C**, The exrepssion of miR-1246 variant in TAMs transfected with miR-1246 inhibitors. **D**, qPCR and western blot showing the level of NLRP3 in M1 macrophages, M2 macrophages and DLD-1-induced TAMs. **E**, ELISA of TNFα and IL1β in

M0 macrophages and induced-TAMs transfected with miR-1246 variant or miR-1246 mimics. **F,** The knockdown and overexpression efficiency of NLRP3 in induced-TAMs. **G**, ELISA of TNFα and IL1β in the CM from TAMs transfected with miR-1246 variant or miR-1246 variant followed by oeNLRP3. **H**, The levels of M2 marker genes in DLD-1-induced TAMs transfected with miR-1246 variant or miR-1246 variant followed by NLRP3 overexpression. **I**, The knockdown and overexpression efficiency of TRAF6 in induced-TAMs. **J**, The expression of M1 and M2 marker genes in induced-TAMs transfected with siTRAF6. **K**, The Kaplan-Meier plot of NLRP3 and TRAF3 in READ patients. Bar graphs show mean + SD. **/***/****P< 0.01/0.001/0.0001.

Table. S1

Dataset1	
16 upregulated miRNAs in both patient samples and cell line media	8 downregulated miRNAs after primary tumor resection
hsa-let-7a	hsa-let-7a
hsa-miR-1224-5p	hsa-miR-1224-5p
hsa-miR-1229	hsa-miR-1229
hsa-miR-1246	hsa-miR-1246
hsa-miR-1268	hsa-miR-150
hsa-miR-1290	hsa-miR-21
hsa-miR-1308	hsa-miR-223
hsa-miR-150	hsa-miR-23a
hsa-miR-181b	
hsa-miR-181d	
hsa-miR-1915	
hsa-miR-21	
hsa-miR-223	
hsa-miR-23a	
hsa-miR-483-5p	
hsa-miR-638	

Dataset2	
18 upregulated miRNAs in the serum of patients with liver metastasis	
hsa-miR-1288	hsa-miR-761
hsa-miR-1204	hsa-miR-4478

hsa-miR-4437	
hsa-miR-23a	
hsa-miR-548v	
hsa-miR-302c	
hsa-miR-642b	
hsa-miR-3618	
hsa-miR-16	
hsa-miR-19a	
hsa-miR-19b	
hsa-miR-1246	
hsa-miR-3681	
hsa-miR-92a	
hsa-miR-320a	
hsa-miR-3150a-5p	

Table. S2

miR-1246 variant	Abundance
ACTIN1	82133131
HNRNPUL1	44305732
HNRNPA2B1	25311214
DHB12	14987791
METK2	95201665
RS30	64962732
DDX7	57159773
SSB	55621191
SDPR	53233183
RBMX	37101437
STAU1	33441555

Table. S3

GeneName	log2FC	Pvalue	padj	Case1	Case1	Case2	Case2
ARG1	-5.3067569	0.05307718	1	0	0	14	20
CCL20	-1.8861821	3.27E-09	1.10E-07	86	94	637	460
CCL2	-0.1125	0.31236906	0.58741797	4588	3854	7160	7666
CXCL12	-0.398733	0.07910055	0.25507218	336	325	738	686
CCL24	-4.8083251	0.12309944	1	0	0	10	7
CXCL13	0.39238596	0.28299469	0.5566876	4136	4612	4277	6455
KLF4	-0.3148395	0.2738305	0.54534433	209	203	447	392
CSF1	-0.0661719	0.58114493	0.80032089	1798	1611	3219	2633

CD86	0.8812188	5.46E-13	3.03E-11	2115	2042	1894	1792
CCL5	0.89004871	5.88E-16	4.30E-14	3385	2885	2992	2546
CXCL10	3.22112809	5.90E-117	6.25E-114	3776	3312	575	659
CD74	1.00328702	2.95E-22	3.22E-20	7415	6828	6870	4839
IDO1	2.33455428	6.92E-13	3.76E-11	486	300	134	120
CD40	0.99064521	3.28E-05	0.00049675	702	477	529	441
CXCL8	-1.156614	3.61E-34	5.79E-32	8972	7517	35108	25379
CCL8	2.41231714	3.09E-114	2.73E-111	5437	4962	1583	1600
CD163	0.9005113	1.41E-28	1.91E-26	6385	6220	5948	5114
IL1B	-0.8441611	3.69E-22	3.94E-20	5066	4375	15445	12358
PTGS2	-1.3959214	0.00132527	0.01161172	76	48	296	238
IL23A	-0.8009695	0.14164691	1	50	60	115	193

Table. S4

Target gene	3P-seq tags + 5	Total sites	7merm8 sites	7mer-A1 sites	6mer sites	Representative miRNA	Cumulative weighted context	Total context++ score
NLRP3	5	1	1	0	1	hsa-miR-1246	-0.13	-0.13
TNFAIP3	150	1	0	1	1	hsa-miR-1246	-0.05	-0.05
TRAF6	91	1	1	0	1	hsa-miR-1246	-0.01	-0.04

Table. S5

qPCR primers

Gene name	Forward (5’-3’)	Reverse (5’-3’)
h-ALOX15	GGAGCCTTCCTAACCTACAGC	CGATTCCTTCCACATACCGA
h-IL10	AACCAAGACCCAGACATCAAG	TTTCACAGGGAAGAAATCGA
h-CD163	ACAATGTTCAGTGTCCAAAAGG	TCGTCCAAGTCCCAAGAGTC
h-TGFB1	GCAGCACGTGGAGCTGTA	CAGCCGGTTGCTGAGGTA

h-GAPDH	GGAGCGAGATCCCTCCAAAAT	GGCTGTTGTCATACTTCTCATGG
h-IL1B	CTAAACAGATGAAGTGCTCC	GGTCATTCTCCTGGAAGG
h-IL8	ACAGCAGAGCACACAAGCTTC	ATCAGGAAGGCTGCCAAGAG
m-ALOX15	GGCTCCAACAACGAGGTCTAC	AGGTATTCTGACACATCCACCTT
m-IL10	GCTCTTACTGACTGGCATGAG	CGCAGCTCTAGGAGCATGTG
m-CD163	ATGGGTGGACACAGAATGGTT	CAGGAGCGTTAGTGACAGCAG
m-TGFB1	CTCCCGTGGCTTCTAGTGC	GCCTTAGTTTGGACAGGATCTG
m-IL1B	GCAACTGTTCCTGAACTCAACT	ATCTTTTGGGGTCCGTCAACT
m-MCP1	GCTACAAGAGGATCACCAGCAG	GTCTGGACCCATTCCTTCTTGG
h-NLRP3	GGACTGAAGCACCTGTTGTGCA	TCCTGAGTCTCCCAAGGCATTC
h-TNFAIP3	CTCAACTGGTGTCGAGAAGTCC	TTCCTTGAGCGTGCTGAACAGC
h-TRAF6	CAATGCCAGCGTCCCTTCCAAA	CCAAAGGACAGTTCTGGTCATGG
h-TUT7	ATAACACCAGGGAACTATGGGA	CATTCATCCAAGCGGGTTGAC
h-TUT4	TGCTCAACAGGTGGCTGGTTCA	GAGTTCTGTGGAAATGGCTGAGG
h-SNRPB	TTGGCACCTTCAAGGCTTTTGAC	AGACCGAGGACTCGCTTCTCTT
h-hnRNPA2B1	ATTGATGGGAGAGTAGTTGAGCC	AATTCCGCCAACAAACAGCTT
h-DIS3L2	CATGTCCGACAAGCTGACCTTC	CGCAGGGATTTTCTCAGTTGGG
h-METTL3	AAGGAACTCGTTGAGGCTGA	CACCTGTGTGGAGACAATGG
h-Oct4	GTCCGAGTGTGGTTCTGTA	CTCAGTTTGAATGCATGGGA

microRNA detection

	Forward (5’-3’)	Reverse (5’-3’)
miR-1246 (RNU2-1f)	GCGCGATGGATTTTTGGAGCAG	
miR-23a	ATCACATTGCCAGGGATT	
U6	CTCGCTTCGGCAGCACA	AACGCTTCACGAATTTGCGT

Table. S6

Antibodies

Protein name	Catalog Numbers	Applications
GAPDH	Abcam, ab8245	WB, 1:1000
Alix	CST, #92880	WB, 1:1000
TSG101	CST, #72312	WB, 1:1000
CD81	CST, #10037	WB, 1:1000
Calnexin	CST, #2679	WB, 1:1000
METTL3	Abcam, ab195352	WB, 1:1000
Oct4	Abcam, ab181557	WB, 1:1000
TUT7	Sigma, HPA020620	WB, 1:500
TUT4	Abcam, ab89165	WB, 1:1000
DIS3L2	Abcam, ab181473	WB, 1:500
SNRPB	Abcam, ab155026	WB, 1:2000
hnRNPA2B1	Abcam, ab31645	WB, 1:1000; IP
MVP	Abcam, ab273093	WB, 1:1000
YBX1	CST, #4202	WB, 1:1000
NLRP3	CST, #15101	WB, 1:1000
TNFAIP3	CST, #5630	WB, 1:1000
TRAF6	Abcam, ab33915	WB, 1:4000
CD86	CST, #91882	IHC, 1:100
CD86	BD Pharmingen, 555660	Flow cytometry, 1:100
CD206	CST, #24595	IHC, 1:200
CD206	BD Pharmingen, 555954	Flow cytometry, 1:100

Table. S7 siRNA/shRNA sequence

**Name**	**Sequence (5′ to 3′)**
si-hnRNPA2B1-1	GGAGCCTTCCTAACCTACAGC
si-hnRNPA2B1-2	GCAAGACCUCAUUCAAUUGTT
si-hnRNPA2B1-3	GGCUUUGUCUAGACAAGAAAU
siTUT4-1	GGUUGCUUCAGACUUUAUA
siTUT4-2	GAAAAGAGGCACAAGAAAA
siTUT4-3	GAAUGUUAUUUGUGAAGAAAG
siTUT7-1	GAAAAGAGGCACAAGAAAA
siTUT7-2	GCAAAGAGGACAAAGAAAU
siTUT7-3	GGACCUAGAAUUCCUGUUAUA
siRNU2-1	CUUAUCAGUUUAAUAUCU
siRNU2-1	GUAUCUGUUCUUAUCAGU
shMETTL3-1	GGGCCCAAGTGCAAGAATTCT
shMETTL3-2	GCTGCACTTCAGACGAATT
shMETTL3-3	CAGTGGATCTGTTGTGATA
shPOU5F1-1	GGCCACACGTAGGTTCTTGAA
shPOU5F1-2	AGGTTCTTGAATCCCGAATGG
shPOU5F1-3	GCTTCAAGAACATGTGTAA
siNLRP3-1	GGAUCAAACUACUCUGUGA
siNLRP3-2	GAAGUGGGGUUCAGAUAAU
siNLRP3-3	CGCUAAUGAUCGACUUCAAUG
siTRAF6 -1	GGGUACAAUACGCCUUACATT
siTRAF6 -2	UGUAAGGCGUAUUGUACCCTT
siTRAF6-3	GGCAGACUGUGACACUCAAUU
siDIS3L2-1	CAAACUUAGCUACGAGCAU
siDIS3L2-2	AGGAGGAGUCUGACGGUGA
siDIS3L2-3	GUAGUUAAACCAGAGAGCA

siSNRPB-1	CAAGCCAAAGAACUCCAAA
siSNRPB-2	GGACCUCCUCCCAAAGAUA
siSNRPB-3	GGACCUCCUCCCAAAGAUACU

Sequences for microRNA mimics and inhibitors

miR-1246 mimics	AAUGGAUUUUUGGAGCAGG
miR-1246 variant	AAUGGAUUUUUGGAGCAGGGAGA
miR-1246 inhibitors	CCUGCUCCAAAAAUCCAUU
Scramble control	ACUAGUCGAUCUAUGUGUGAUATT

Table. S8

RNU2-1 sequence
ATCGCTTCTC GGCCTTTTGG CTAAGATCAA GTGTAGTATC TGTTCTTATC AGTTTAATAT CTGATACGTC CTCTATCCGA GGACAATATA TTAAATGGAT TTTTGGAGCA GGGAGATGGA ATAGGAGCTT GCTCCGTCCA CTCCACGCAT CGACCTGGTA TTGCAGTACC TCCAGGAACG GTGCACCC

Table. S9 Patient information

Samples	Gender	Age	CEA	CA19-9	Tumor site	Date of operation	size (cm)	T	N	M	Vascular invasion (negative 0; positive 1)	Nerve invasion (negative 0; positive 1)
1	male	64	9.94	6.22	rectum	1/16/18	5.5	3	2a	x	1	0
2	female	53	4.33	45.11	rectum	1/17/18	2.8	3	2a	x	1	0
3	female	65	1.91	0.63	ascending colon	1/17/18	5.5	3	1a	x	1	0
4	male	64	5.11	18.29	rectum	1/17/18	3.8	3	2b	x	1	1
5	female	77	30.08	71.57	sigmoid colon	2018/1/18	5	4	2a	1	0	1
6	male	63	2.64	0.6	rectum	2/28/18	5	3	0	x	0	0
7	female	46	2.1	5.53	rectum	3/2/18	3	1	0	x	0	0
8	male	26	2.59	5.55	rectum	3/2/18	5	3	2b	x	1	1
9	male	53	1.57	7.56	rectum	3/5/18	3	3	0	x	0	0
10	male	49	7.98	0.9	rectum	3/21/18	3	2	0	x	0	0
11	male	58	2.62	11.37	rectum	2018/3/23	3	3	0	x	0	0
12	female	85	2.47	20.11	ascending colon	5/3/18	5.5	3	0	x	0	0
13	male	62	3.62	26.79	rectum	5/3/18	3.2	1	0	x	0	0
14	female	64	13.59	53.27	sigmoid colon	6/20/18	3.7	4	0	x	0	0
15	male	57	18.23	31.41	rectum	2018/6/20	3.5	3	2a	x	0	0
16	male	62	20.92	19.16	descending colon	2018/6/21	4.5	4	1b	x	0	0
17	male	67	3.69	10.23	ascending colon	2018/6/21		3	0	x	0	0
18	female	66	113	84.89	sigmoid colon	7/16/18	4.6	4	2b	x	1	1
19	male	62	9.2	6.03	rectum	2018/7/16	2	3	0	x	0	0
20	male	75	17.3	214.9	ascending colon	7/17/18	4.8	3	0	x	0	0
21	male	28	4.29	1.33	rectum	2018/7/17	6	3	1c	1	0	0
22	male	26	16.16	116.2	ascending colon	7/17/18	7	4	1a	x	0	0
23	male	77	2.33	6.26	sigmoid colon	8/22/18	2.5	3	0	x	0	0
24	male	60	7.11	16.35	rectum	2018/8/23	4.5	3	2a	x	1	0
25	male	58	5.07	518.7	ascending colon	8/23/18	3	3	1a	1	0	0
26	male	52	15.03	251.5	transverse colon	8/24/18	8.5	3	1b	x	0	0
27	male	75	8.04	16.9	descending colon	2018/8/27	3	4	0	x	0	0
28	male	49	0.53	8.73	rectum	2018/9/25	2.7	2	0	x	0	0
29	male	35	4.96	177.3	rectum	2018/9/26	6	3	0	x	0	0
30	male	61	28.11	1.01	sigmoid colon	2018/9/27	2.7	3	1b	x	0	1
31	female	71	8.79	15.14	descending colon	2018/9/28	4.3	4	1a	x	0	0
32	female	78	3.6	1.62	descending colon	2018/9/28	4	3	0	x	0	0
33	female	72	3.48	9.4	sigmoid colon	2018/9/28	4	3	1b	x	0	1

34	female	72	1.94	6.65	sigmoid colon	2019/1/16	4.5	3	1b	x	1	x
35	male	63	1.61	10.64	rectum	2019/1/16	2.5	1	0	x	x	x
36	male	63	1.99	13.8	sigmoid colon	2019/1/18	5	3	2a	x	x	x
37	female	83	3.67	16.52	rectum	2019/1/18	3	2	0	x	x	x
38	female	55	2.38	19.54	ascending colon	2019/1/18	6.2	3	1b	x	x	1
39	female	74	22.77	41.61	ascending colon	2019/3/26	3.5	4	2b	x	1	1
40	male	37	94.38	0.78	rectum	2019/3/26	7	3	0	x	x	x
41	male	73	3.7	7.99	rectum	2019/3/27	3.5	3	0	x	x	x
42	female	66	56.61	31.3	sigmoid colon	2019/3/27	5.5	4	1b	1	x	x
43	female	59	24.88	33.56	rectum	2019/5/14	4.5	3	1b	x	x	x
44	male	56	2.53	12.98	sigmoid colon	2019/5/14	6	2	2a	x	x	x
45	female	68	5.44	18.19	rectum	2019/5/14	2.5	2	1c	x	x	x
46	male	69	3.14	10.86	rectum	2019/5/14	5	3	0	x	x	x
47	female	69	27.57	15.47	ascending colon	2019/6/19	8	3	0	x	x	x
48	female	47	89.29	1.27	rectum	2019/6/21	4.5	3	1a	x	x	x
49	female	38	2.38	28.11	rectum	2019/6/21	2.2	2	0	x	x	x
50	male	84	1.88	3.45	ascending colon	2019/6/24	3.5	3	1b	x	x	x
51	male	66	4.3	43.93	rectum	2019/7/30	2	2	1b	1	x	x
52	male	73	3.4	11.59	sigmoid colon	2019/7/30	4	3	0	x	x	x
53	male	53	1.62	31.1	ascending colon	2019/7/30	7	3	0	1	x	x
54	female	61	2.17	14.89	sigmoid colon	2019/7/30	5	4	2b	x	1	1
55	female	65	2.7	5.39	ascending colon	2019/7/30	7	3	1a	x	x	x
56	female	64	4.24	25.15	ascending colon	2019/7/30	4	3	1b	x	x	x
57	male	68	3.35	22.65	rectum	2019/8/13	1.5	2	0	x	x	x
58	male	83	5.02	34.21	rectum	2019/8/13	3	3	1c	x	x	1
59	male	54	6.34	13.9	rectum	2019/8/13	3.8	2	0	x	x	x
60	female	70	5.36	<0.600	ascending colon	2019/8/15	7	3	1a	x	x	x
